# Depressive symptoms during early adulthood and the development of physical multimorbidity in the UK: an observational cohort study

**DOI:** 10.1016/S2666-7568(21)00259-2

**Published:** 2021-12

**Authors:** Jorge Arias-de la Torre, Amy Ronaldson, Matthew Prina, Faith Matcham, Snehal M Pinto Pereira, Stephani L Hatch, David Armstrong, Andrew Pickles, Matthew Hotopf, Alex Dregan

**Affiliations:** a Institute of Psychiatry, Psychology and Neuroscience, King's College London, London, UK; b ESRC Centre for Society and Mental Health, King's College London, London, UK; c Department of Primary Care and Public Health Sciences, King's College London, London, UK; d CIBER Epidemiology and Public Health (CIBERESP), Madrid, Spain; e Institute of Biomedicine, University of Leon, Leon, Spain; f Institute of Sport, Exercise and Health, Faculty of Medical Sciences, University College London, London, UK; g South London and Maudsley NHS Foundation Trust, London, UK

## Abstract

**Background:**

An understanding of whether early-life depression is associated with physical multimorbidity could be instrumental for the development of preventive measures and the integrated management of depression. We therefore aimed to map out the cumulative incidence of physical multimorbidity over adulthood, and to determine the association between the presence of depressive symptoms during early adulthood and the development of physical multimorbidity in middle age.

**Methods:**

In this observational cohort study, we used pooled data from the 1958 National Child Development Study (NCDS) and the 1970 British Cohort Study (BCS). Cohort waves were pooled in each decade of adult life available (when cohort members were aged 26 years in the BCS and 23 years in the NCDS [baseline]; 34 years in the BCS and 33 years in the NCDS [age 34 BCS/33 NCDS]; 42 years in the BCS and NCDS [age 42 BCS/NCDS]; and 46 years in the BCS and 50 years in the NCDS [age 46 BCS/50 NCDS]). We included participants who had completed the nine-item Malaise Inventory at baseline, and did not have a history of physical multimorbidity, any physical multimorbidity at baseline, or the presence of depressive symptoms before the development of physical multimorbidity. The presence of depressive symptoms was determined using the nine-item Malaise Inventory (cutoff score ≥4). Physical multimorbidity was defined as having at least two measures of any of the following ten self-reported groups of long-term conditions: asthma or bronchitis; backache; bladder or kidney conditions; cancer; cardiovascular conditions; convulsions or epilepsy; diabetes; hearing conditions; migraine; and stomach, bowel, or gall conditions. Cumulative incidence (with 95% CI) of physical multimorbidity was calculated for each decade considered after baseline, with physical multimorbidity being assessed as both a dichotomous and categorical variable. The association between depressive symptoms and the development of physical multimorbidity was assessed using adjusted relative risk ratios (with 95% CIs).

**Findings:**

Analyses included 15 845 participants, of whom 4001 (25·25%; 95% CI 24·57–25·93) had depressive symptoms at baseline and 11 844 (74·75%; 74·07–75·42) did not. The cumulative incidence of physical multimorbidity (dichotomous) ranged over the study period from 2263 (18·44%; 95% CI 17·75–18·14) of 12 273 participants at age 34 BCS/33 NCDS, to 4496 (42·90%; 41·95–43·85) of 10 481 participants at age 46 BCS/50 NCDS, and was consistently higher in participants with depressive symptoms at baseline. The adjusted relative risk of physical multimorbidity was higher in participants with depressive symptoms than in those without and remained stable over the study period (adjusted relative rate ratio 1·67, 95% CI 1·50–1·87, at age 34 BCS/33 NCDS; 1·63, 1·48–1·79, at age 42 BCS/NCDS; and 1·58, 1·43–1·73, at age 46 BCS/50 NCDS).

**Interpretation:**

The presence of depressive symptoms during early adulthood is associated with an increased risk of the development of physical multimorbidity in middle age. Although further research about the drivers of this relationship is needed, these results could help to enhance the integrated management of individuals with depressive symptoms and the development of preventive strategies to reduce the effect and burden of physical multimorbidity.

**Funding:**

UK Medical Research Council and Guy's Charity.

## Introduction

Physical multimorbidity can be defined as the coexistence of two or more long-term physical conditions.^1^, ^2^ Several studies suggest that disorders with similar underlying pathology tend to appear together, and that depression is the mental disorder most frequently present in patients with physical multimorbidity.^3^, ^4^ A large 2018 study examining dyad and triad combinations of chronic conditions showed that patients with multimorbidity and depression reported a consistently poorer quality of life than those without depression.^5^ Additionally, several studies have shown that multimorbidity patterns comprising concurrent depression and physical disorders are associated with higher premature mortality.^6^, ^7^

**Research in context**

**Evidence before this study**

Previous studies suggest that depression is the mental disorder most frequently present in patients with multimorbidity. Moreover, multimorbidity patterns comprising concurrent depression and physical disorders are associated with higher premature mortality. Unlike most long-term physical conditions, the peak of depression onset is during adolescence and early adult life (15–30 years) and, hence, it might represent an important risk marker or common risk factor for physical multimorbidity. As has been previously proposed, the presence of depressive symptoms in young adults could be associated with the incidence of physical multimorbidity in middle age. However, this evidence was obtained from studies focused on samples mainly composed of older adults (≥65 years), with specific characteristics (eg, only women) or that had short follow-up time.

**Added value of this study**

To our knowledge, this is one of the first population-wide studies assessing the effect of depressive symptoms on the development of physical multimorbidity from early adulthood over three decades. It overcomes some of the limitations of previous research (eg, cross-sectional design, small sample size, or short follow-up time) as we used pooled data from two large and population-based cohorts from the UK: the 1958 National Child Development Study and the 1970 British Cohort Study. Our results show that approximately a quarter of the UK population included in these cohorts had depressive symptoms during early adulthood. Furthermore, the cumulative incidence of physical multimorbidity continuously increased over the study period, being consistently higher in participants with depressive symptoms during early adulthood than in those without. In addition, it was found that the development of physical multimorbidity was more likely among participants with depressive symptoms during early adulthood than in those without, but the risk of physical multimorbidity in those with depressive symptoms compared with those without remained stable.

**Implications of all the available evidence**

This study highlights the relevance of depressive symptoms during early adulthood as an important risk factor for the development of physical multimorbidity in middle age. Although further research about the drivers of this relationship is needed, these results indicate that early intervention strategies for those who develop depressive symptoms early in adulthood could serve to reduce the risk of developing multimorbidity as they get older.

Unlike long-term physical conditions, the peak of depression onset is during adolescence and early adult life (15–30 years), and, hence, it might represent an important risk marker or common risk factor for both specific physical disorders and physical multimorbidity.^8^ Moreover, the presence of depressive symptoms in young adults could be differently related to the incidence of physical multimorbidity at different points over adulthood.^9^, ^10^, ^11^, ^12^ Several studies have highlighted variations by age in physical multimorbidity rates, with a peak in later life (>60 years).^1^, ^6^, ^7^, ^8^ However, most of these studies were focused on samples mainly composed of older adults (≥65 years), had samples with specific characteristics, or had short follow-up time. Therefore, mapping out the cumulative incidence of physical multimorbidity from early adulthood could provide insight into the potential effect of depressive symptoms on its development over the lifespan. This knowledge will strengthen the evidence base to develop targeted preventive interventions and inform the delivery of integrated health-care services, thus helping to reduce the adverse effects of physical multimorbidity related to depression.

The association between previous depressive symptoms and physical multimorbidity might be partly attributable to different behavioural, personal, biological, and lifestyle factors.^13^, ^14^, ^15^ Additionally, as has been consistently found, the effect of both physical and mental disorders is not equally distributed over the social continuum, being more common in lower socioeconomic groups and contributing to inequalities in health.^16^ Consideration of these types of variables when studying physical multimorbidity might help to obtain more accurate estimations of the incidence both of depressive symptoms and physical multimorbidity, thus helping to increase the capability of generalising these estimations to different population groups.

In this study, we aimed to map out the cumulative incidence of physical multimorbidity over adulthood and to determine the associations between the presence of depressive symptoms during early adulthood and the development of physical multimorbidity later on in life.

## Methods

### Study design and population

We did an observational cohort study based on pooled data from the 1958 National Child Development Study (NCDS) and the 1970 British Cohort Study (BCS), following the STROBE guidelines. The NCDS included 17 415 children born in England, Scotland, and Wales in a single week of 1958 who will be followed up during their entire life. From the original population, around 12 000 cohort members (about 69% of the original sample) are still participating in the study.^17^ 9137 participants (about 76% of the original sample) contributed in the last wave in 2013 (when participants were aged 55 years). The BCS is similar in design to the NCDS and included a cohort of 17 287 children born in a single week of 1970 in the UK. BCS cohort members will also be followed up during their entire life. More than 9500 participants (about 55% of the original sample) contributed data in the last wave in 2016 (when participants were aged 46 years).^18^ Data collected in the BCS are similar to those collected in the NCDS, facilitating cross-cohort comparisons and pooling. Both cohorts provide comprehensive data on physical and mental health, health-care use, lifestyle, education, employment, family life, and socioeconomic circumstances. Additionally, both cohorts have a non-response rate lower than 5% (4·1% for the BCS and 1·2% for the NCDS) at the age of 0 of the cohort members. When cohort members were aged 26 years, the non-response rate was 31·7% in the BCS and 24·1% in the NCDS. To enhance the robustness of the conclusions and avoid potentially spurious associations caused by small sample sizes (particularly of those individuals with a large number of long-term conditions and depressive symptoms), data from BCS and NCDS were pooled within each decade of adult life, as recommended by previous research on harmonisation of mental health measures within UK cohort studies.^19^ Data were pooled considering the closest waves as possible: baseline (when cohort members were aged 26 years in the BCS and 23 years in the NCDS [age 26 BCS/33 NCDS]); 34 years in the BCS and 33 years in the NCDS (age 34 BCS/33 NCDS); 42 years in the BCS and NCDS (age 42 BCS/NCDS); and 46 years in the BCS and 50 years in the NCDS (age 46 BCS/50 NCDS). The baseline point was selected to be consistent with the outcome measure: the nine-item version of the Malaise Inventory.^20^ This questionnaire was consistently captured from this point onwards and has shown a suitable comparability between the BCS and the NCDS.^19^ For data pooling and to try to minimise possible heterogeneity in the results, only variables captured in the same way (or those that could be transformed to have the same values or categories) in the pooled waves and in both cohorts were considered. More detailed information about the cohorts, including the original non-response rate to the survey (at the week of birth), and the specific questions for each wave, cohort, and condition can be found on the website of the Centre for Longitudinal Studies at University College London (London, UK). We included participants who completed the nine-item Malaise Inventory at baseline and in each decade of life considered. Participants with a history of physical multimorbidity or a physical multimorbidity at baseline, and participants who developed a physical multimorbidity before depressive symptoms after baseline were excluded from analyses.

### Study variables

The outcome of this study was physical multimorbidity, defined as the coexistence of two or more self-reported long-term physical conditions.^17^, ^18^ Physical conditions were identified according to the participants self-response to specific questions, such as “Have you ever had diabetes?” followed by “Have you seen a doctor for diabetes in the last 12 months?” or “Whether diabetes was diagnosed by a doctor”. Although the actual format of the questions varied within surveys over time and between cohorts (appendix pp 1–4), they were asked in the same format for each condition and assessed the same construct (disease), enabling comparison of physical multimorbidity patterns over time. The following ten self-reported groups of long-term conditions (not individual diseases) included in at least two follow-up waves of both the BCS and NCDS were included in the physical multimorbidity measure: asthma or bronchitis; backache; bladder or kidney conditions; cancer; cardiovascular conditions; convulsions or epilepsy; diabetes; hearing conditions; migraine; and stomach, bowel, or gall conditions. These conditions were selected because of their consistent inclusion over waves in both cohorts, particularly among the last waves. Because these diseases could be considered long-term conditions, when one of them was reported once by a cohort member it was assumed that it remained throughout the study period. The presence of physical multimorbidity was considered both as a dichotomous variable (yes *vs* no) and as categorical variable (no physical multimorbidity, two conditions, three conditions, or four or more conditions).

Caseness for depressive symptoms during early adulthood was determined using the nine-item version of the Malaise Inventory.^20^ The Malaise Inventory is a self-reported psychological distress tool included in both the BCS and NCDS to determine levels of psychological distress or depressive symptoms in the general population.^20^, ^21^ The nine-item version of the Malaise Inventory incorporates nine dichotomous items (yes *vs* no) of 24 items of the original version (appendix p 5).^20^, ^21^, ^22^ The nine-item version was consistently captured across all surveys since young adulthood (ie, the baseline wave) in both the BCS and NCDS, thus allowing the data pooling.^19^, ^23^ In addition, measurement invariance for the nine-item version of the Malaise Inventory was supported across cohorts, and therefore it could reliably be compared in terms of covariances and means across cohorts and age groups.^19^, ^23^ The nine-item Malaise Inventory final score is computed by adding the scores of all items, offering a score that ranges from 0 to 9. For this study, the cutoff point for depressive symptoms was a score of 4 or more, as suggested by previous research and to improve the comparability with their results.^22^, ^24^

Because of their relationship with both the presence of depressive symptoms and physical multimorbidity, the following baseline socioeconomic characteristics and health-related and behavioural variables were considered in this study: gender (male and female), ethnicity (White, mixed, Asian, Black, and other), employment status (working, unemployed, full-time education, long-standing sick or disability, housework, and other situations such as incarceration or long holidays), marital status (single; married; and widowed, separated, or divorced), drinking alcohol (most days, one or two times per week, less often than one or two times per week, special occasions, and never), and smoking status (smoker, never smoked, and ex-smoker). Additionally, the familial socioeconomic status of cohort members was considered (from I, the most advantaged group, to V, the most disadvantaged social group). Familial socioeconomic status was considered as the highest level from the father, mother, or both when cohort members were aged 10 years in the BCS and 11 years in the NCDS based on the occupational social class 1970 classification.^17^, ^18^

### Data analysis

To account for missing data in the main analysis, we carried out multiple imputation using chained equations with ten imputations. Due to the potential heterogeneity between the two studies arising from generational differences and possible cohort effects, we considered which specific cohort the individual belonged to during the analyses as an additional covariate. All outcome variables, the presence of depressive symptoms at baseline, all baseline characteristics, and the specific cohort to which the participants belong were included in the multiple imputation model. After imputation, descriptive analysis of the baseline characteristics of the study population was done using absolute and relative frequencies (n and %) together with 95% CIs both overall and according to the presence of depressive symptoms.

To evaluate longitudinal associations between the presence of depressive symptoms in early adulthood (ie, baseline) and the development of physical multimorbidity, we calculated the cumulative incidence of physical multimorbidity (95% CI) for each pooled wave after baseline both overall and separately for participants with and those without depressive symptoms at baseline. Furthermore, to assess the magnitude of the relationship between the presence of depressive symptoms at baseline and the cumulative incidence of physical multimorbidity, multinomial logistic regression models were fitted considering physical multimorbidity as the outcome and depressive symptoms at baseline as the main explanatory factor. From these models, we obtained crude relative risk ratios, adjusted relative risk ratios, and their 95% CIs, for participants with depressive symptoms at baseline versus those without (reference). Models were adjusted for all baseline characteristics (without considering potential changes in these characteristics across surveys over time) because of their clinical relevance by use of an a priori theory-driven approach (while trying to be as parsimonious and replicable as possible), and the absence of interactions between the presence of depressive symptoms at baseline and all of the covariates was verified.

In sensitivity analyses, we calculated the cumulative incidence of physical multimorbidity separately for each of the pooled cohorts, and we examined the relationship between the presence of depressive symptoms at baseline and the development of physical multimorbidity. To observe the performance of the cutoff score of 4 or more of the nine-item Malaise Inventory, the main analyses were replicated using cutoff scores of 3 or more and 5 or more. Additionally, we did a post-hoc sensitivity analysis to assess the relationship between depressive symptoms at baseline and the development of physical multimorbidity by gender and parental socioeconomic group. For the sensitivity analyses, to capture the socioeconomic group of the participants and ensure comparability between the BCS and NCDS data, the General Register Office for England and Wales 1970 classification categories were collapsed into non-manual and manual. We used the National Statistics Socio-economic Classification criteria to classify participants into non-manual (supervisory to managerial work: categories 1, 2, and 3 non-manual) and manual (routine or semi-routine work: categories 3 non-manual, 4, and 5) occupations. Furthermore, the frequencies of the specific conditions among individuals with physical multimorbidity were considered as sensitivity analyses and an analysis excluding the two most frequent conditions (backache and migraine) was carried out. Because of the potential differences with the rest of the cohort members, we also did sensitivity analyses calculating the cumulative incidence of physical multimorbidity during childhood (when cohort members were aged 10 years in the BCS and 11 years in the NCDS) and adolescence (when cohort members were aged 16 years in both the BCS and NCDS) and their relationship with the presence of depressive symptoms at baseline. At baseline and during adolescence, the presence of physical multimorbidity was determined from a self-reported questionnaire. In childhood, physical multimorbidity was determined from a parental questionnaire (mainly answered by the mother) and by a medical examination made to about half of participants of both cohorts. In addition, we calculated the prevalence of physical multimorbidity at baseline, both overall and stratified by cohort and depressive symptoms. For these analyses, all individuals with information on depressive symptoms at baseline and who completed the questionnaires during childhood, adolescence, or both were considered. Finally, we did a sensitivity analysis including in the physical multimorbidity measures only those individuals who reported each condition at least two times during follow-up to observe differences by depressive symptoms in loss to follow-up. All analyses were carried out using Stata (version 16).

### Role of the funding source

The funder of the study had no role in study design, data collection, data analysis, data interpretation, or writing of the report.

## Results

**Figure 1:**
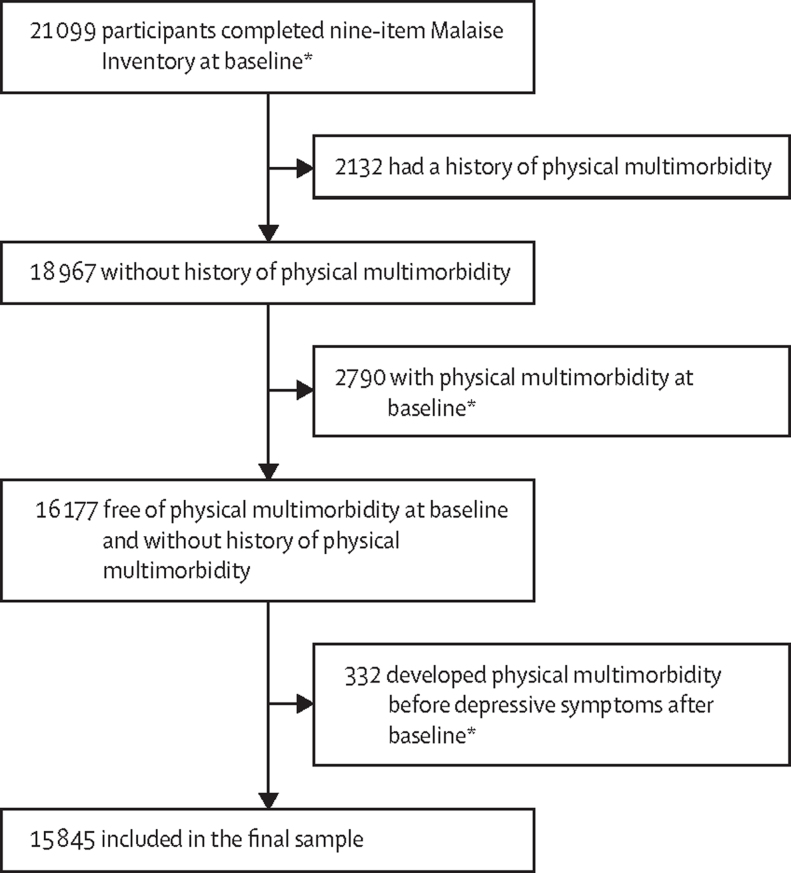
Sample determination *Baseline was defined as cohort member age of 26 years in the British Cohort Study and 23 years in the National Child Development Study.

All participants in the BCS and NCDS who completed the nine-item Malaise Inventory at baseline (cohort member age 26 years in the BCS and 23 years in the NCDS) and the decades following were included (21 099 [97·95%] of all 21 448 participants). After excluding individuals with a history of physical multimorbidity, those with physical multimorbidity at baseline, and those who developed physical multimorbidity before depressive symptoms, 15 845 individuals were included in the final sample (figure 1).

4001 (25·25%; 95% CI 24·57–25·93) of 15 845 participants had depressive symptoms at baseline: 10 465 (66·05%; 65·32–66·79) participants were from NCDS 1958 and 5380 (33·95%; 33·21–34·68) were from BCS 1970 (table 1). 11 844 (74·75%; 95% CI 74·07–75·42) participants did not have depressive symptoms at baseline. Most participants were White and working at baseline, and there were differences in the distribution of all variables except ethnicity and some categories of marital status between participants with and those without depressive symptoms at baseline (table 1). The percentage of missing data for the covariates at baseline ranged from 0 (gender and cohort) to 22·59% (familial socioeconomic status).

**Table 1:** General baseline characteristics overall and by depressive symptoms in the study population

	**All participants (n=15 845)**	**Participants without depression (n=11 844)**	**Participants with depression (n=4001)**
**Cohort**			
1958 NCDS	66·05% (65·32–66·79)	66·69% (65·84–67·54)	64·16% (62·67–65·64)
1970 BCS	33·95% (33·21–34·69)	33·31% (32·46–34·16)	35·84% (34·36–37·33)
**Gender**			
Male	50·56% (49·79–51·34)	51·94% (51·04–52·84)	46·49% (44·94–48·03)
Female	49·44% (48·66–50·21)	48·06% (47·16–48·96)	53·51% (51·97–55·06)
**Ethnicity**			
White	97·88% (97·62–98·14)	97·91% (97·61–98·20)	97·81% (97·30–98·32)
Mixed	0·31% (0·21–0·41)	0·27% (0·16–0·38)	0·42% (0·19–0·65)
Asian	0·86% (0·70–1·02)	0·78% (0·60–0·96)	1·09% (0·73–1·46)
Black	0·38% (0·27–0·49)	0·41% (0·28–0·54)	0·29% (0·10–0·48)
Other	0·57% (0·44–0·70)	0·63% (0·47–0·79)	0·38% (0·16–0·60)
**Employment status**			
Working	77·71% (77·06–78·36)	79·62% (78·90–80·35)	72·03% (70·64–73·43)
Unemployed	7·44% (7·03–7·85)	6·96% (6·50–7·42)	8·88% (7·99–9·76)
Full-time education	2·68% (2·42–2·93)	2·97% (2·67–3·27)	1·81% (1·40–2·23)
Long-standing sick or disability	5·65% (4·48–6·82)	3·14% (2·12–4·15)	1·31% (0·96–1·65)
Housework	9·88% (9·41–10·35)	8·46% (7·96–8·96)	14·10% (13·01–15·18)
Other situations*	1·73% (1·52–1·93)	1·68% (1·45–1·91)	1·87% (1·45–2·29)
**Marital status**			
Single	56·95% (56·18–57·72)	57·48% (56·58–58·37)	55·39% (53·85–56·94)
Married	39·71% (38·95–40·47)	39·70% (38·82–40·57)	39·74% (38·22–41·26)
Widowed, separated, or divorced	3·34% (3·06–3·62)	2·82% (2·53–3·12)	4·87% (4·20–5·54)
**Familial socioeconomic status**			
I	6·72% (6·25–7·19)	7·30% (6·73–7·87)	5·07% (4·30–5·84)
II	27·53% (26·74–28·33)	28·72% (27·77–29·67)	24·15% (22·66–25·63)
III (non-manual†)	20·91% (20·19–21·63)	21·38% (20·51–22·25)	19·58% (18·21–20·96)
III (manual†)	30·50% (29·68–31·32)	29·25% (28·28–30·21)	34·06% (32·48–35·65)
IV	11·75% (11·14–12·35)	11·06% (10·41–11·70)	13·70% (12·47–14·93)
V	2·59% (2·28–2·91)	2·30% (1·96–2·63)	3·44% (2·77–4·11)
**Alcohol consumption**			
Most days	23·67% (23·01–24·33)	24·52% (23·74–25·29)	21·17% (19·89–22·44)
One to two times per week	44·69% (43·91–45·46)	45·60% (44·70–46·50)	41·99% (40·46–43·52)
Less often than one to two times per week	15·65% (15·08–16·21)	15·07% (14·42–15·71)	17·36% (16·18–18·54)
Special occasions	11·81% (11·32–12·32)	11·08% (10·52–11·65)	13·96% (12·89–15·04)
Never	4·18% (3·87–4·50)	3·73% (3·39–4·08)	5·52% (4·81–6·23)
**Smoking status**			
Yes	38·24% (37·48–39·00)	35·76% (34·90–36·63)	45·57% (44·02–47·11)
No never	37·41% (36·66–38·17)	39·10% (38·22–39·98)	32·44% (30·98–33·89)
Ex-smoker	24·35% (23·68–25·02)	25·14% (24·36–25·92)	21·99% (20·71–23·28)

Data are estimates (95% CI [logit-transformed]) obtained after multiple imputation. Depressive symptoms at baseline (cohort member age 26 years in the BCS and 23 years in the NCDS) were assessed using the nine-item Malaise Inventory with a cutoff score of 4 or more. BCS=British Cohort Study. NCDS=National Child Development Study.

* Other situations including incarceration, long holidays, unemployed people who do not want to work, and other very infrequent situations.

† Non-manual is defined as supervisory to managerial occupations, and manual is defined as routine or semi-routine occupations, based on the National Statistics Socio-economic Classification criteria.

**Figure 2:**
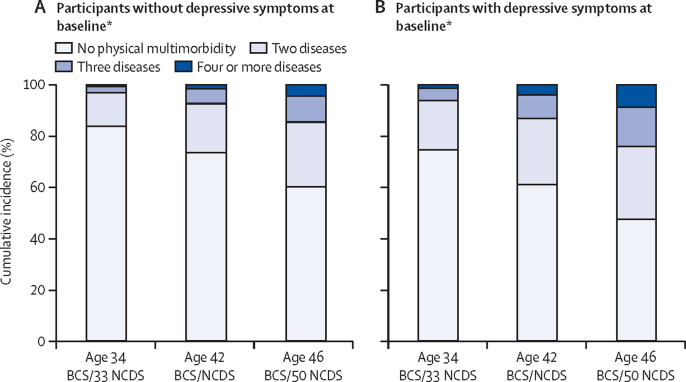
Cumulative incidence of physical multimorbidity over pooled waves BCS=British Cohort Study. NCDS=National Child Development Study. *Baseline was defined as cohort member age of 26 years in the BCS and 23 years in the NCDS.

For the whole population, the cumulative incidence of physical multimorbidity increased from 2263 (18·44%; 95% CI 17·75–18·14) of 12 273 participants at age 34 BCS/33 NCDS, to 4496 (42·90%; 41·95–43·85) of 10 481 participants at age 46 BCS/50 NCDS, and was consistently higher in those with depressive symptoms at baseline than in those without (table 2). When physical multimorbidity was taken as a categorical variable and assessed over time, it showed a continuous increase, particularly in participants with four or more diseases who had depressive symptoms at baseline (figure 2).

**Table 2:** Cumulative incidence of physical multimorbidity overall and stratified by the presence of depressive symptoms at baseline

		**Age 34 BCS/33 NCDS (n=12 273)**		**Age 42 BCS/NCDS (n=12 022)**		**Age 46 BCS/50 NCDS (n=10 481)**	
		n	% (95% CI)	n	% (95% CI)	n	% (95% CI)
**All participants**							
Baseline depressive symptoms positive		3038	24·75% (23·99–25·52)	2983	24·81% (24·04–25·60)	2548	24·31% (23·49–25·14)
Physical multimorbidity (dichotomous)		2263	18·44% (17·75–18·14)	3551	29·54% (28·72–30·36)	4496	42·90% (41·95–43·85)
Physical multimorbidity (categorical)							
	No physical multimorbidity (no or one disease)	10 010	81·56% (80·09–82·24)	8471	70·46% (69·64–71·27)	5985	57·10% (56·15–58·05)
	Two diseases	1797	14·64% (14·02–15·24)	2501	20·80% (20·08–21·54)	2731	26·06% (25·22–26·91)
	Three diseases	382	3·11% (2·81–3·44)	800	6·65% (6·21–7·11)	1202	11·47% (10·86–12·09)
	Four or more diseases	84	0·68% (0·54–0·85)	250	2·08% (1·83–2·35)	563	5·37% (4·95–5·82)
**Participants without depressive symptoms**							
Physical multimorbidity (dichotomous)		1495	16·19% (15·44–16·96)	2394	26·48% (25·58–27·41)	3159	39·82% (38·74–40·91)
Physical multimorbidity (categorical)							
	No physical multimorbidity (no or one disease)	7740	83·81% (83·04–84·56)	6645	73·51% (72·59–74·42)	4774	60·18% (59·09–61·25)
	Two diseases	1214	13·15% (12·46–13·85)	1736	19·21% (18·40–20·03)	2008	25·31% (24·36–26·28)
	Three diseases	232	2·51% (2·20–2·85)	521	5·76% (5·29–6·26)	811	10·22% (9·56–10·91)
	Four or more diseases	49	0·53% (0·39–0·70)	137	1·52% (1·27–1·79)	340	4·29% (3·85–4·75)
**Participants with depressive symptoms**							
Physical multimorbidity (dichotomous)		768	25·28% (23·84–26·87)	1157	38·79% (37·03–40·53)	1337	52·47% (50·51–54·42)
Physical multimorbidity (categorical)							
	No physical multimorbidity (no or one disease)	2270	74·72% (73·13–76·25)	1826	61·21% (59·44–62·97)	1211	47·53% (45·57–49·48)
	Two diseases	583	19·19% (17·80–20·63)	765	25·65% (24·08–27·25)	723	28·38% (26·63–30·17)
	Three diseases	150	4·94% (4·19–5·77)	279	9·35% (8·33–10·46)	391	15·35% (14·00–16·80)
	Four or more diseases	35	1·15% (0·80–1·60)	113	3·79% (3·12–4·54)	223	8·75% (7·68–9·92)

Depressive symptoms at baseline (cohort member age 26 years in the BCS and 23 years in the NCDS) assessed using the nine-item Malaise Inventory with a cutoff score of 4 or more. BCS=British Cohort Study. NCDS=National Child Development Study.

Irrespective of whether it was presented as a dichotomous or ordered categorical variable, a higher risk of physical multimorbidity was observed at all timepoints in participants with depressive symptoms at baseline than in those without (table 3). The magnitude of the risk of incident physical multimorbidity (dichotomous) in participants with depressive symptoms remained stable over the study period (adjusted relative risk ratio 1·67, 95% CI 1·50–1·87, at age 34 BCS/33 NCDS; 1·63, 1·48–1·79, at age 42 BCS/NCDS; and 1·58, 1·43–1·73, at age 46 BCS/50 NCDS; table 3). Taking physical multimorbidity as an ordered categorical variable, the same pattern of slightly decreased risk was observed for all physical multimorbidity categories (table 3).

**Table 3:** Relationship between depressive symptoms at baseline and the development of physical multimorbidity over adulthood

	**Age 34 BCS/33 NCDS (n=12 273)**		**Age 42 BCS/NCDS (n=12 022)**		**Age 46 BCS/50 NCDS (n=10 481)**	
	Relative risk ratio (95% CI)	Adjusted relative risk ratio (95% CI)	Relative risk ratio (95% CI)	Adjusted relative risk ratio (95% CI)	Relative risk ratio (95% CI)	Adjusted relative risk ratio (95% CI)
**Physical multimorbidity (dichotomous)**						
No physical multimorbidity (no or one disease)	1 (ref)	1 (ref)	1 (ref)	1 (ref)	1 (ref)	1 (ref)
Yes	1·75 (1·59–1·93)	1·67 (1·50–1·87)	1·75 (1·61–1·92)	1·63 (1·48–1·79)	1·67 (1·53–1·83)	1·58 (1·43–1·73)
**Physical multimorbidity (categorical)**						
No physical multimorbidity (no or one disease)	1 (ref)	1 (ref)	1 (ref)	1 (ref)	1 (ref)	1 (ref)
Two diseases	1·64 (1·47–1·83)	1·61 (1·43–1·82)	1·60 (1·45–1·77)	1·52 (1·37–1·69)	1·42 (1·28–1·58)	1·38 (1·23–1·54)
Three diseases	2·20 (1·79–2·82)	1·89 (1·49–2·20)	1·95 (1·67–2·27)	1·76 (1·50–2·08)	1·90 (1·66–2·18)	1·77 (1·53–2·05)
Four or more or more diseases	2·44 (1·57–3·77)	2·13 (1·25–3·65)	3·00 (2·32–3·87)	2·44 (1·86–3·20)	2·59 (2·16–3·10)	2·27 (1·87–2·75)

Depressive symptoms at baseline (cohort member age 26 years in the BCS and 23 years in the NCDS) assessed using the nin-item Malaise Inventory with a cutoff score of 4 or more. BCS=British Cohort Study. NCDS=National Child Development Study.

*Adjusted for gender, ethnicity, employment status, marital status, familial socioeconomic status, alcohol consumption, smoking status, and cohort.

In sensitivity analyses, when stratified by cohort, the cumulative incidence of physical multimorbidity was higher in the NCDS cohort than in the BCS cohort over the study period (appendix p 6). The associations between baseline depressive symptoms and the incidence of physical multimorbidity were significant in all cases except in the BCS at age 34 BCS/33 NCDS in participants with three conditions, and the association was not possible to calculate in those with four or more conditions (appendix p 7). Considering the associations found using cutoff scores of 3 or more and 5 or more in the nine-item Malaise Inventory, although higher and lower in magnitude when using a cutoff score of 5 or more and 3 or more, respectively, the patterns of risk found were similar to using a cutoff score of 4 or more (appendix p 8). Depressive symptoms at baseline were more frequent in women (appendix p 9) and in participants belonging to non-manual social classes (appendix p 10). Additionally, the risk of incident physical multimorbidity in participants with depressive symptoms was also higher than in those without in these groups (appendix pp 9–10). Considering the specific conditions, backache and migraine were the conditions most frequently reported, and cancer and diabetes (but convulsions or epilepsy at age 46 BCS/50 NCDS) were less frequently reported, in participants with physical multimorbidity over the study period (appendix p 11). With exclusion of backache and migraine, the cumulative incidence of physical multimorbidity was lower than when they were included, but the magnitude of the relationship found between participants with depressive symptoms at baseline and the incidence of physical multimorbidity was similar (appendix p 12).

Sensitivity analyses to determine the effect of physical multimorbidity during childhood and adolescence on the development of depressive symptoms at baseline showed that the risk of depressive symptoms at baseline was higher in participants with physical multimorbidity than in those without (appendix p 13). Additionally, these analyses showed that although the proportion of individuals with physical multimorbidity was higher during adolescence than during childhood, the risk of depressive symptoms in those with physical multimorbidity in adolescence was similar (appendix p 13). The prevalence of depressive symptoms and physical multimorbidity at baseline (before exclusion of participants with physical multimorbidity at baseline, those with a history of physical multimorbidity, and those with physical multimorbidity before depressive symptoms after baseline) was higher in the BCS than in the NCDS (appendix p 14). Furthermore, physical multimorbidity was more prevalent in participants with depressive symptoms at baseline than in those without, independently of the cohort (appendix p 14). In the sensitivity analysis that included in the physical multimorbidity measures only those individuals who reported each condition at least two times during follow-up, although the patterns found were similar to those who reported the conditions one or more times, the risk of incident physical multimorbidity was higher in those who reported each condition at least two times during follow-up (appendix p 15). Finally, the percentage of individuals lost to follow-up was similar but slightly higher in those with depressive symptoms than in those without (appendix p 16). All full models are included in the appendix (pp 17–26).

## Discussion

This study analysed the effect of depressive symptoms during early adulthood on the development of physical multimorbidity in middle age using pooled data from two large population-based cohorts from the UK. The results show that the development of physical multimorbidity in middle age is more likely in individuals with depressive symptoms during early adulthood than in those without. Furthermore, a slight decrease in the magnitude of this relationship over time was found, particularly among participants with fewer conditions. Although new research about the drivers of this relationship is needed, these results highlight the importance of considering the presence of depressive symptoms during early adulthood as a relevant risk factor for the development of physical multimorbidity in middle age and the potential benefits of taking the presence of depressive symptoms into account when planning preventive strategies and integrated care interventions against the development of physical multimorbidity.

Several studies have estimated the prevalence and trajectories of different chronic diseases and multimorbidity.^25^, ^26^, ^27^, ^28^, ^29^, ^30^ Our results show a higher prevalence of both physical multimorbidity and the specific conditions than found in previous studies.^29^ However, methodological (eg, number of diseases) and sampling (eg, age and country) differences between previous research and our study make it difficult to directly compare the findings. In a 2019 systematic review, the multimorbidity rate at 50 years of age varied from 30% to 50% among men and from 40% to 60% among women, placing our study finding (43%) within both ranges.^29^ Likewise, an earlier review indicated a multimorbidity prevalence of less than 20% before the age of 40 years in the general population, which is in line with our study overall finding (18%) during the third decade of life.^31^ Yet, among participants with depression, we observed higher rates of physical multimorbidity (25%) at ages younger than 40 years, which might be due to variation in the number and nature of conditions or sample characteristics between our study and previous research. Nevertheless, this review did not include UK-based evidence, and between-country variation might explain our finding of a higher rate of physical multimorbidity in participants with depression than in those without. Additionally, the more stringent definition criteria for a chronic condition used in previous studies^31^ (eg, duration of ≥3 months and relevant medication) compared with our study might also explain the observed differences. Besides, studies in primary care populations tended to report lower prevalence rates than those in the general population due to the more severe nature of conditions diagnosed in clinical settings. Thus, it is possible that our study included a larger number of people with mild to moderately severe conditions. This suggestion is in line with evidence based on the sensitivity analysis that excluded backache and migraine, which revealed reduced incidence of physical multimorbidity, similar to previous studies.^29^ Because low back pain is among the leading global causes of years lived with disability,^32^, ^33^ and migraine is one of the most frequent and comorbid neurological disorders,^34^ their inclusion in this study's physical multimorbidity measure might encourage similar inclusion in future studies.

Our study overcomes some of the limitations of some previous research (eg, cross-sectional designs, focus on specific populations or diseases, small sample sizes, and short follow-up time),^8^, ^9^, ^10^, ^11^, ^12^ and it includes a UK population-wide sample of young adults without physical multimorbidity who were followed up for more than 50 years. In line with previous research using more selected samples and with shorter follow-up time,^9^, ^11^ our results show that the presence of depressive symptoms is prospectively related to the development of physical multimorbidity in middle age. Additionally, sensitivity analyses showed that the frequency of both depressive symptoms and physical multimorbidity and the magnitude of their relationship were higher in women and in participants from lower socioeconomic status. Different behavioural and biological factors have been suggested as possible drivers of this relationship.^2^, ^8^, ^13^, ^35^ Additionally, although their influence is not completely clear, we were unable to adjust our model for some factors that might have had an effect, including biological factors (eg, obesity, particularly among middle-aged women, and inflammation), life events (eg, related to personal relationships), and, particularly, early-life events such as the potential exposure to an unhealthy environment during childhood and adolescence.^8^, ^35^, ^36^, ^37^ However, although the relationship of these factors with both depression and physical multimorbidity could be relevant, albeit complex, to the best of our knowledge, few studies and datasets capture all (or most) of them from early-life stages. One example of this complexity could be the possible role of inflammation as a mediator in the relationship between depression and physical multimorbidity that, as suggested by previous evidence,^38^ might be affected by different psychosocial and behavioural factors. Therefore, further research considering trajectories of depressive symptoms from childhood, taking into account the covariates considered, early-life circumstances, and the previously mentioned factors (eg, of obesity), as well as their possible role and potential variation over time, will be highly valuable to have an in-depth understanding of the drivers of the relationships found between the presence of depressive symptoms and the incidence of physical multimorbidity.

The slight decrease in the magnitude of the relationship between depressive symptoms and the risk of physical multimorbidity should also be noted. These findings might be explained by changes in psychosocial factors related to ageing, such as changes in self-perceived wellbeing or life satisfaction, and by biological factors including inflammation. As suggested by previous research,^39^, ^40^ adverse early-life circumstances might be related to disease accumulation, but this could be attenuated by later-life circumstances and experiences. Nevertheless, biological factors such as high levels of pro-inflammatory cytokines, might play a relevant part in the relationship between acute effects of depressive symptoms and physical multimorbidity.^41^, ^42^

Our study has several limitations. Depression itself could cause physical symptoms that might affect the self-reported conditions of physical multimorbidity. We must also point out the limitation related to the nature of the data used to create the physical multimorbidity measure. The physical multimorbidity measure included wide groups of conditions and specific symptoms and not specific diseases, which could lead to higher rates of physical multimorbidity than even in some previous studies that have included a greater number of conditions in the physical multimorbidity measure.^30^ Additionally, it should be noted that these conditions and symptoms, were self-reported and not considered from diagnostic codes based on standardised clinical criteria (eg, from International Classification of Diseases codes) and that the exact diagnostic date is not captured. Hence, the conditions could be considered in a different way by participants, and a time-to-event analysis could have limitations. Despite these limitations, our study relied on multiple data sources (eg, self-reports, medical examination, validated depression tools, and biomedical data) collected by trained interviewers, including nurses, and the included conditions were reflective of the most common patient-reported disorders at different life stages.^17^, ^18^ A further limitation relates to the assumption that the physical conditions reported remain throughout the study period. Although the findings of the sensitivity analyses validated the robustness of physical multimorbidity diagnosis, a single-report approach to define specific health conditions might have overestimated physical multimorbidity rates, particularly in older ages. Moreover, although primarily data-driven, our physical multimorbidity measure included most conditions that should be part of any multimorbidity measure (eg, diabetes, cardiovascular, cancer, kidney, and sensory problems).^43^ Another possible limitation relates to the use of the nine-item version of the Malaise Inventory instead of the full version (24 items) or its full psychological subscale (15 items). Nevertheless, the nine-item version is the most commonly used version of the Malaise Inventory,^21^ it is captured in most of the follow-up waves of both the BCS and NCDS,^19^ and its use will allow future intra-cohort and inter-cohort comparisons because it is included also in other cohort studies, such as the Millennium cohort study.^22^ Limitations related to data pooling must be mentioned. Differences between cohorts in the prevalence of physical multimorbidity were found, and this prevalence was consistently higher in the NCDS than in the BCS, possibly caused by generational changes such as changes in habits and lifestyles. Additionally, the follow-up was not exactly at the same age in some waves in the different cohorts, and not all the conditions considered were included in all waves for each cohort. However, the cohort to which the participant belonged was included in all models and, thus, the potential effect that it could have on the estimates could be considered, at least partially, controlled. Furthermore, the comparability of the Malaise Inventory between cohorts is suitable, and pooling cohort data^19^, ^23^ allowed us to have a larger sample size, enhancing the power to detect potentially relevant relationships in less frequent groups (eg, those with four or more diseases), improving the robustness of results. This could also enhance the representativeness of the study sample, as it allowed us to include different generations within the study thereby accounting for potential generational differences. Finally, it should be mentioned that the participation in these cohorts is voluntary,^17^, ^18^ and that participants are being followed up over their whole lives. Thus, the studies risk healthy volunteer bias, with those with the worst physical and mental health possibly declining to participate, which might affect the data.

In conclusion, the results of the study provide evidence that depressive symptoms during early adulthood are a risk factor for the development of physical multimorbidity in middle age. New studies about this relationship considering trajectories of depressive symptoms from childhood and different psychosocial and biological factors, such as changes in life satisfaction and inflammation from early adulthood, will help to elucidate its drivers and help us to understand underlying mechanisms. Taking the effect of depressive symptoms on the development of physical multimorbidity into account could be relevant for the integrated management of individuals and aid the development of preventive strategies.

## Data sharing

Data from both the BCS and NCDS are publicly available for research purposes under request to the UK Data Service.

## Declaration of interests

MH is the principal investigator of the RADAR-CNS programme, a precompetitive public private partnership funded by the Innovative Medicines Initiative and European Federation of Pharmaceutical Industries and Associations. The programme receives support from Janssen, Biogen, Merck Sharp & Dohme, UCB, and Lundbeck. All other authors declare no competing interests.
