# Supplementary material for: Depressive symptoms during early adulthood and the development of physical multimorbidity in the UK: an observational cohort study
Source: Lancet Healthy Longev. 2021 Dec;2(12):e801–10. doi: 10.1016/S2666-7568(21)00259-2 (PMC8636278; doi:10.1016/S2666-7568(21)00259-2)
Supplement: Supplementary appendix [file mmc1.pdf]

THE LANCET
Healthy Longevity

Supplementary appendix

This appendix formed part of the original submission and has been peer reviewed. We post it as supplied by the authors.

Supplement to: Arias-de la Torre J, Ronaldson A, Prina M, et al. Depressive symptoms during early adulthood and the development of physical multimorbidity in the UK: an observational cohort study. *Lancet Healthy Longev* 2021; **2:** e801–10.

Appendix

Questions were asked in each wave to account for the conditions included in the physical multimorbidity measure at baseline (age 26 from BCS and age 23 from NCDS).

Condition	BCS	NCDS
Asthma or chronic bronchitis	Have you suffered from any of these Since you were 16? Asthma<br>Have you suffered from any of these Since you were 16? Chronic bronchitis	Since your sixteenth birthday have you had any attack of asthma or wheezy bronchitis?
Backache	Have you suffered from any of these Since you were 16? Persistent joint or back pain	NI
Bladder or kidney conditions	Have you suffered from any of these Since you were 16? Bladder or kidney problems	What is the nature of the child's handicap or disability? Disorder of Kidney or urinary tract*
Cancer	Have you suffered from any of these Since you were 16? Cancer	What is the nature of the child's handicap or disability? (Malignancy) including Leukaemia*
Cardiovascular conditions	Has this teenager ever had any of the following conditions? Pathological hearth condition*	What is the nature of the child's handicap or disability? Heart condition*
Convulsions or epilepsy	Have you suffered from any of these Since you were 16? Fits, convulsions, epilepsy	Since your sixteenth birthday have you had any kind of first, convulsions, long faints or loss of consciousness?
Diabetes	Have you suffered from any of these Since you were 16? Diabetes	What is the nature of the child's handicap or disability? Diabetes*
Hearing conditions	Have you suffered from any of these Since you were 16? Hearing difficulties	What is the nature of the child's handicap or disability? Hearing defect*
Migraine	Have you suffered from any of these Since you were 16? Migraine	Since your sixteenth birthday have suffered from migraine or recurrent sick headaches?
Stomach/bowel/gall conditions	Have you suffered from any of these Since you were 16? Stomach or other digestive problems	NI

*BCS: British Cohort Study; NCDS: National Child Development Study.NI: Not included in this or the previous wave; *: condition considered from the previous wave (age 16 both BCS and NCDS) to have a cohort free of pMM.*

Questions asked to account for the conditions included in the physical multimorbidity measure at age 34 BCS and 33 NCDS.

Condition	BCS	NCDS
Asthma or chronic bronchitis	Since [date of last interview / January 2000] have you had any of the health problems listed on this card? Asthma or wheezy bronchitis	Have you suffered from, or been told you had bronchitis in the last twelve months?<br>Over the past twelve months have you had any attacks of wheezing or asthma so severe that you could speak only one or two words at a time between breaths?
Backache	Since [date of last interview / January 2000] have you had any of the health problems listed on this card? Recurrent backache, prolapsed disc, sciatica	Have you ever had back pain lasting for more than one day at any time in the past 12 months?
Bladder or kidney conditions	NI	Have you suffered from, or been told you had kidney/bladder trouble in the last twelve months?
Cancer	Since [date of last interview / January 2000] have you had any of the health problems listed on this card? Cancer	Have you suffered from, or been told you had cancer in the last twelve months?
Cardiovascular conditions	Since [date of last interview / January 2000] have you had any of the health problems listed on this card? High blood pressure	Have you suffered from, or been told you had high blood pressure in the last twelve months?<br>Have you suffered from, or been told you had hearth trouble in the last twelve months?
Convulsions or epilepsy	Since [date of last interview / January 2000] have you had any of the health problems listed on this card? Convulsion, fit, epileptic seizure	Have you suffered from, or been told you had epilepsy in the last twelve months?
Diabetes	Since [date of last interview / January 2000] have you had any of the health problems listed on this card? (Sugar) Diabetes	Have you suffered from, or been told you had diabetes in the last twelve months?
Hearing conditions	Since [date of last interview / January 2000] have you had any of the health problems listed on this card? Problems with hearing	NI
Migraine	Since [date of last interview / January 2000] have you had any of the health problems listed on this card? Migraine or Leukaemia	Have you suffered from, or been told you had migraine in the last twelve months?
Stomach/bowel/gall conditions	NI	Have you suffered from, or been told you had stomach trouble or indigestion in the last twelve months?<br>Have you suffered from, or been told you had gall bladder trouble in the last twelve months?

BCS: British Cohort Study; NCDS: National Child Development Study; NI: Not included in this wave.

Questions were asked to account for the conditions included in the physical multimorbidity measure at age 42 from BCS and NCDS.

Condition	BCS	NCDS
Asthma or chronic bronchitis	Since [date of last interview / month four years prior to interview] have you had any of the health problems listed on this card? Asthma or wheezy bronchitis	Have you suffered asthma in the last 12 months?<br>Have you suffered bronchitis in the last 12 months?
Backache	Since [date of last interview / month four years prior to interview] have you had any of the health problems listed on this card? Recurrent backache, prolapsed disc, sciatica or other back problem	Have you had back pain, lumbago or sciatica in the past 12 months?
Bladder or kidney conditions	Since [date of last interview / month four years prior to interview] have you had any of the health problems listed on this card? Problems with bladder or kidneys	Have you had bladder or kidney problems in the last 12 months?
Cancer	Since [date of last interview / month four years prior to interview] have you had any of the health problems listed on this card? Cancer or Leukaemia	Have you had cancer in the last 12 months?
Cardiovascular conditions	Since [date of last interview / month four years prior to interview] have you had any of the health problems listed on this card? High blood pressure	Have you had high blood pressure in the last 12 months?
Convulsions or epilepsy	Since [date of last interview / month four years prior to interview] have you had any of the health problems listed on this card? Convulsion, fit, epileptic seizure	Have you had a problem with fits, convulsions or epilepsy in the last 12 months?
Diabetes	Since [date of last interview / month four years prior to interview] have you had any of the health problems listed on this card? (Sugar) Diabetes	Have you had diabetes in the last 12 months?
Hearing conditions	Since [date of last interview / month four years prior to interview] have you had any of the health problems listed on this card? Problems with hearing	NI
Migraine	Since [date of last interview / month four years prior to interview] have you had any of the health problems listed on this card? Migraine	Have you had a migraine in the last 12 months?
Stomach/bowel/gall conditions	Since [date of last interview / month four years prior to interview] have you had any of the health problems listed on this card? Problems with stomach, bowels or gall bladder	Have cohort member had ulcer in last 12 months?<br>Have cohort member had gallstones in last 12 months?<br>Have cohort member had irritable bowel syndrome in last 12 months?<br>Have cohort member had ulcerative colitis in last 12 months?

BCS: British Cohort Study; NCDS: National Child Development Study; NI: Not included in this wave.

Questions were asked to account for the conditions included in the physical multimorbidity measure at age 46 from BCS and age 50 from NCDS.

Condition	BCS	NCDS
Asthma or chronic bronchitis	Since [last interview / four years ago] have you had any of the health problems listed on this card? Asthma or wheezy bronchitis	Interviewer: exclude temporary conditions. Are you currently suffering from any of the health problems listed on this card? Asthma or wheezy bronchitis
Backache	Since [last interview / four years ago] have you had any of the health problems listed on this card? Recurrent backache, prolapsed disc, sciatica or other back problem	Interviewer: exclude temporary conditions. Are you currently suffering from any of the health problems listed on this card? Recurrent backache, prolapsed disc, sciatica
Bladder or kidney conditions	Since [last interview / four years ago] have you had any of the health problems listed on this card? Problems with bladder or kidneys	Interviewer: exclude temporary conditions. Are you currently suffering from any of the health problems listed on this card? Problems with bladder or kidneys
Cancer	Since [last interview / four years ago] have you had any of the health problems listed on this card? Cancer or Leukaemia	Interviewer: exclude temporary conditions. Are you currently suffering from any of the health problems listed on this card? Cancer or Leukaemia
Cardiovascular conditions	Since [last interview / four years ago] have you had any of the health problems listed on this card? High blood pressure	Interviewer: exclude temporary conditions. Are you currently suffering from any of the health problems listed on this card? High blood pressure
Convulsions or epilepsy	Since [last interview / four years ago] have you had any of the health problems listed on this card? Convulsion, fit, epileptic seizure	Interviewer: exclude temporary conditions. Are you currently suffering from any of the health problems listed on this card? Convulsion, fit, epileptic seizure?
Diabetes	Since [last interview / four years ago] have you had any of the health problems listed on this card? (Sugar) Diabetes	Interviewer: exclude temporary conditions. Are you currently suffering from any of the health problems listed on this card? (Sugar) diabetes
Hearing conditions	Since [last interview / four years ago] have you had any of the health problems listed on this card? Problems with hearing	Interviewer: exclude temporary conditions. Are you currently suffering from any of the health problems listed on this card? Problems with hearing
Migraine	Since [last interview / four years ago] have you had any of the health problems listed on this card? Migraine	Interviewer: exclude temporary conditions. Are you currently suffering from any of the health problems listed on this card? Migraine
Stomach/bowel/gall conditions	Since [last interview / four years ago] have you had any of the health problems listed on this card? Problems with stomach, bowels or gall bladder	Interviewer: exclude temporary conditions. Are you currently suffering from any of the health problems listed on this card? Problems with stomach, bowels or gall bladder

BCS: British Cohort Study; NCDS: National Child Development Study; NI: Not included in this wave.

Items included in the 9-item version of the Malaise Inventory.

1. Do you feel tired most of the time?
2. Do you often feel miserable or depressed?
3. Do you often get worried about things?
4. Do you often get into a violent rage?
5. Do you often suddenly become scared for no reason?
6. Are you easily upset or irritated?
7. Are you constantly keyed up and jittery?
8. Does every little thing get on your nerves?
9. Does your heart often race like mad?

Cumulative incidence (%) of physical multimorbidity (pMM) stratified by Cohort.

	Age 34 BCS/33 NCDS		Age 42 BCS/NCDS		Age 46 BCS/50 NCDS	
	**n**	**% (95% CI)**	**n**	**% (95% CI)**	**n**	**% (95% CI)**
NCDS 1958	8,148	-	8,042	-	6,924	-
**Depressive symptoms during early adulthood**	1,953	23.97 (23.05-24.91)	1,924	23.92 (23.00-24.87)	1,610	23.25 (22.26-24.27)
**pMM (dichotomous)**	1,814	22.26 (21.36-23.18)	2,534	31.51 (30.49-32.54)	3,146	45.44 (44.25-44.61)
**pMM (categorical)**						
No pMM (0 or 1 disease)	6,334	77.74 (76.82-78.84)	5,508	68.49 (67.46-69.51)	3,778	54.56 (53.38-55.74)
2 diseases	1,399	17.17 (16.36-18.00)	1,730	21.51 (20.62-22.43)	1,837	26.53 (25.49-27.59)
3 diseases	334	4.10 (3.68-4.55)	608	7.56 (6.99-8.16)	871	12.58 (11.81-13.39)
4 or more diseases	81	0.99 (0.79-1.23)	196	2.44 (2.11-2.80)	438	6.33 (5.76-6.93)
BCS 1970	4,125	-	3,980	-	3,557	-
**Depressive symptoms during early adulthood**	1,085	26.30 (24.96-27.68)	1,059	26.61 (25.24-28.01)	938	26.37 (24.93-27.85)
**pMM (dichotomous)**	449	10.88 (9.95-11.88)	1,017	25.55 (24.20-26.94)	1,350	37.95 (36.35-39.57)
**pMM (categorical)**						
No pMM (0 or 1 disease)	3,676	89.12 (88.12-90.05)	2,963	74.45 (73.06-75.80)	2,207	62.05 (60.42-63.65)
2 diseases	398	9.65 (8.76-10.59)	771	19.37 (18.15-20.63)	894	25.13 (23.61-26.59)
3 diseases	48	1.16 (0.86-1.54)	192	4.82 (4.18-5.54)	331	9.31 (8.37-10.30)
4 or more diseases	3	0.07 (0.02-0.21)	54	1.36 (1.02-1.77)	125	3.51 (2.93-4.17)

Depressive symptoms during early adulthood (age 23 NCDS and 26 BCS) assessed using the Malaise Inventory with a cut-off score 4+; n: number of individuals; % 95% CI: cumulative incidence and 95% Confidence Interval.

Relationship between have depressive symptoms during early adulthood and the development of physical multimorbidity (pMM) over adulthood stratified by cohort

	Age 34 BCS/33 NCDS		Age 42 BCS/NCDS		Age 46 BCS/50 NCDS 50s	
	**RRR (95% CI)**	**aRRR (95% CI)**	**RRR (95% CI)**	**aRRR (95% CI)**	**RRR (95% CI)**	**aRRR (95% CI)**
NCDS 1958						
**pMM (dichotomous)**						
No pMM (0 or 1 disease)	1.00	1.00	1.00	1.00	1.00	1.00
Yes	1.87 (1.67-2.10)	1.74 (1.53-1.98)	1.83 (1.64-2.03)	1.63 (1.43-1.86)	1.74 (1.55-1.94)	1.61 (1.43-1.82)
**pMM (categorical)**						
No pMM (0 or 1 disease)	1.00	1.00	1.00	1.00	1.00	1.00
2 diseases	1.72 (1.52-1.95)	1.67 (1.45-1.93)	1.64 (1.45-1.86)	1.50 (1.32-1.72)	1.47 (1.28-1.68)	1.42 (1.23-1.63)
3 diseases	2.42 (1.93-3.04)	1.97 (1.53-2.55)	2.08 (1.74-2.49)	1.81 (1.50-2.19)	1.94 (1.65-2.29)	1.77 (1.48-2.11)
4 or more diseases	2.42 (1.54-3.78)	2.01 (1.17-3.47)	2.88 (2.15-3.85)	2.25 (1.66-3.06)	2.64 (2.14-3.26)	2.24 (1.79-2.80)
BCS 1970						
**pMM (dichotomous)**						
No pMM (0 or 1 disease)	1.00	1.00	1.00	1.00	1.00	1.00
Yes	1.65 (1.34-2.03)	1.50 (1.20-1.87)	1.69 (1.45-1.97)	1.64 (1.39-1.93)	1.62 (1.39-1.89)	1.52 (1.29-1.78)
**pMM (categorical)**						
No pMM (0 or 1 disease)	1.00	1.00	1.00	1.00	1.00	1.00
2 diseases	1.60 (1.28-1.99)	1.48 (1.17-1.87)	1.57 (1.32-1.86)	1.56 (1.29-1.88)	1.38 (1.16-1.64)	1.33 (1.10-1.60)
3 diseases	1.78 (0.99-3.22)	1.73 (0.78-3.01)	1.75 (1.29-2.38)	1.64 (1.18-2.27)	1.96 (1.53-2.50)	1.78 (1.38-2.31)
4 or more diseases	-	-	3.99 (2.32-6.87)	3.34 (1.79-6.21)	2.84 (1.97-4.10)	2.40 (1.62-3.56)

Depressive symptoms during early adulthood (age 23 NCDS and 26 BCS) assessed using the Malaise Inventory with a cut-off score 4+; RRR: Relative Risk Ratio; 95% CI: 95% Confidence Interval. aRRR: Relative Risk Ratio adjusted for gender, ethnicity, employment status, marital status, familial socioeconomic status, alcohol consumption, smoking status and cohort.

Relationship between have depressive symptoms during early adulthood (Malaise) and the development of physical multimorbidity (pMM) over adulthood.

	Age 34 BCS/33 NCDS (n=12,273)			Age 42 BCS/NCDS (n=12,022)			Age 46 BCS/50 NCDS (n=10,481)		
	**% Depression**	**RRR (95% CI)**	**aRRR (95% CI)**	**% Depressi on**	**RRR (95% CI)**	**aRRR (95% CI)**	**% Depression**	**RRR (95% CI)**	**aRRR (95% CI)**
MI cut-off score 3+									
**pMM (dichotomous)**									
No pMM (0 or 1 disease)	28.85	1.00	1.00	27.40	1.00	1.00	25.50	1.00	1.00
Yes	41.01	1.71 (1.56-1.88)	1.65 (1.48-1.83)	39.71	1.75 (1.61-1.90)	1.63 (1.49-1.78)	36.50	1.68 (1.54-1.82)	1.61 (1.47-1.75)
**pMM (categorical)**									
No pMM (0 or 1 disease)	28.85	1.00	1.00	52.38	1.00	1.00	25.50	1.00	1.00
2 diseases	38.84	1.57 (1.41-1.74)	1.54 (1.37-1.74)	37.19	1.57 (1.43-1.72)	1.48 (1.34-1.64)	32.63	1.41 (1.28-1.56)	1.37 (1.23-1.52)
3 diseases	48.69	2.34 (1.91-2.87)	2.14 (1.70-2.69)	43.88	2.07 (1.79-2.40)	1.93 (1.65-2.25)	39.52	1.91 (1.68-2.17)	1.84 (1.60-2.11)
4 or more diseases	52.38	2.71 (!.76-4.17)	2.11 (1.24-3.60)	51.60	2.83 (1.19-3.64)	2.33 (1.78-3.05)	48.85	2.79 (2.34-3.32)	2.59 (2.14-3.12)
MI cut-off score 5+									
**pMM (dichotomous)**									
No pMM (0 or 1 disease)	3.71	1.00	1.00	3.07	1.00	1.00	2.84	1.00	1.00
Yes	8.26	2.34 (1.95-2.81)	2.09 (1.69-2.59)	6.96	2.36 (1.97-2.82)	2.01 (1.66-2.45)	6.05	2.20 (1.81-2.68)	1.88 (1.52-2.32)
**pMM (categorical)**									
No pMM (0 or 1 disease)	3.71	1.00	1.00	3.07	1.00	1.00	2.84	1.00	1.00
2 diseases	7.35	2.06 (1.58-2.63)	1.93 (1.52-2.45)	5.84	1.96 (1.59-2.41)	1.77 (1.41-2.21)	4.50	1.61 (1.27-2.04)	1.45 (1.12-1.87)
3 diseases	10.47	3.04 (2.16-4.28)	2.61 (1.73-3.93)	8.50	2.93 (2.22-3.87)	2.75 (1.73-3.19)	7.74	2.87 (2.21-3.72)	2.49 (1.88-3.30)
4 or more diseases	17.86	5.65 (3.20-9.97)	3.53 (1.62-7.69)	13.20	4.80 (3.26-7.07)	3.27 (2.12-5.05)	9.95	3.78 (2.76-5.18)	2.84 (1.85-3.77_

Depressive symptoms during early adulthood (age 23 NCDS and 26 BCS); %: percentage with depressive symptoms during early adulthood; RRR: Relative Risk Ratio; 95% CI: 95% Confidence Interval. aRRR: Relative Risk Ratio adjusted for ethnicity, employment status, marital status, familial socioeconomic status, alcohol consumption, smoking status and cohort.

Relationship between have depressive symptoms during early adulthood (Malaise) and the development of physical multimorbidity (pMM) over adulthood by gender.

	**age 34 BCS/33 NCDS (n=12,273)**			**Age 42 BCS/NCDS (n=12,022)**			**Age 46 BCS/50 NCDS (n=10,481)**		
	**% Depression**	**RRR (95% CI)**	**aRRR (95% CI)**	**% Depression**	**RRR (95% CI)**	**aRRR (95% CI)**	**% Depression**	**RRR (95% CI)**	**aRRR (95% CI)**
Men									
**pMM (dichotomous)**									
No pMM (0 or 1 disease)	21.34	1.00	1.00	20.38	1.00	1.00	19.37	1.00	1.00
Yes	29.03	1.51 (1.30-1.75)	1.43 (1.21-1.70)	27.77	1.50 (1.31-1.72)	1.46 (1.27-1.68)	25.60	1.43 (1.25-1.64)	1.41 (1.22-1.62)
**pMM (categorical)**									
No pMM (0 or 1 disease)	21.34	1.00	1.00	20.38	1.00	1.00	19.37	1.00	1.00
2 diseases	27.56	1.40 (1.19-1.66)	1.38 (1.15-1.68)	26.07	1.38 (1.18-1.60)	1.37 (1.17-1.61)	23.11	1.25 (1.07-1.46)	1.25 (1.06-2.48)
3 diseases	35.50	2.03 (1.47-2.80)	1.61 (1.12-2.34)	32.45	1.88 (1.48-2.38)	1.69 (1.32-2.18)	29.08	1.71 (1.40-2.09)	1.69 (1.36-2.09)
4 or more diseases	32.14	1.75 (0.89-3.87)	1.64 (1.61-4.35)	31.40	1.79 (1.12-2.84)	1.74 (1.08-2.80)	30.96	1.87 (1.40-2.49)	1.72 (1.25-2.34)
Women									
**pMM (dichotomous)**									
No pMM (0 or 1 disease)	24.02	1.00	1.00	22.81	1.00	1.00	21.16	1.00	1.00
Yes	37.87	1.93 (1.69-2.20)	1.87 (1.62-2.17)	36.29	1.93 (1.72-2.17)	1.78 (1.57-2.02)	33.33	1.86 (1.64-2.11)	1.73 (1.52-1.98)
**pMM (categorical)**									
No pMM (0 or 1 disease)	24.02	1.00	1.00	22.81	1.00	1.00	21.16	1.00	1.00
2 diseases	36.44	1.81 (1.57-2.10)	1.81 (1.54-2.12)	34.25	1.76 (1.54-2.01)	1.66 (1.44-1.91)	29.51	1.56 (1.35-1.80)	1.49 (1.28-1.74)
3 diseases	42.25	2.31 (1.75-2.10)	2.09 (1.52-2.87)	36.66	1.96 (1.60-2.40)	1.85 (1.49-2.29)	35.50	2.05 (1.71-2.47)	1.88 (1.54-2.29)
4 or more diseases	46.43	2.74 (1.61-4.65)	2.38 (1.22-4.61)	52.44	3.73 (2.72-5.11)	2.95 (2.10-4.13)	45.99	3.17 (2.51-4.02)	2.80 (2.18-3.61)

Depressive symptoms during early adulthood (age 23 NCDS and 26 BCS); %: percentage with depressive symptoms; RRR: Relative Risk Ratio; 95% CI: 95% Confidence Interval. aRRR: Relative Risk Ratio adjusted for ethnicity, employment status, marital status, familial socioeconomic status, alcohol consumption, smoking status and cohort.

Relationship between have depressive symptoms during early adulthood (Malaise) and the development of physical multimorbidity (pMM) over adulthood by parental Familial Socioeconomic Status.

	age 34 BCS/33 NCDS (n=12,273)			Age 42 BCS/NCDS (n=12,022)			Age 46 BCS/50 NCDS (n=10,481)		
	**% Depression**	**RRR (95% CI)**	**aRRR (95% CI)**	**% Depression**	**RRR (95% CI)**	**aRRR (95% CI)**	**% Depression**	**RRR (95% CI)**	**aRRR (95% CI)**
Non manual									
**pMM (dichotomous)**									
No pMM (0 or 1 disease)	22.07	1.00	1.00	20.90	1.00	1.00	19.93	1.00	1.00
Yes	30.34	1.54 (1.31-1.80)	1.53 (1.29-1.82)	29.69	1.60 (1.39-1.83)	1.54 (1.34-1.77)	27.47	1.52 (1.33-1.74)	1.51 (1.31-1.74)
**pMM (categorical)**									
No pMM (0 or 1 disease)	22.07	1.00	1.00	20.90	1.00	1.00	19.93	1.00	1.00
2 diseases	29.18	1.45 (1.22-1.73)	1.48 (1.22-1.78)	27.70	1.45 (1.24-1.70)	1.44 (1.22-1.68)	24.39	1.30 (1.10-1.52)	1.30 (1.10-1.53)
3 diseases	34.56	1.86 (1.30-2.67)	1.74 (1.18-2.56)	31.89	1.77 (1.38-2.27)	1.67 (1.30-2.15)	30.02	1.72 (1.39-2.13)	1.70 (1.36-2.13)
4 or more diseases	41.67	2.52 (1.12-5.69)	1.93 (0.73-5.10)	44.57	3.04 (2.00-4.62)	2.45 (1.57-3.81)	39.61	2.64 (1.97-3.54)	2.56 (1.83-3.44)
Manual									
**pMM (dichotomous)**									
No pMM (0 or 1 disease)	27.16	1.00	1.00	26.52	1.00	1.00	24.92	1.00	1.00
Yes	41.47	1.90 (1.62-2.22)	1.81 (1.52-2.15)	38.95	1.77 (1.54-2.04)	1.66 (1.45-1.92)	35.04	1.62 (1.40-1.88)	1.52 (1.30-1.78)
**pMM (categorical)**									
No pMM (0 or 1 disease)	27.16	1.00	1.00	26.52	1.00	1.00	24.92	1.00	1.00
2 diseases	39.70	1.77 (1.48-2.10)	1.75 (1.45-2.12)	36.62	1.60 (1.36-1.88)	1.52 (1.29-1.79)	31.57	1.39 (1.17-1.66)	1.34 (1.12-1.61)
3 diseases	46.57	2.34 (1.67-2.27)	1.95 (1.34-2.85)	40.86	1.91 (1.50-2.45)	1.82 (1.41-2.34)	36.30	1.72 (1.37-2.15)	1.63 (1.29-2.06)
4 or more diseases	54.54	3.22 (1.61-6.41)	2.80 (1.25-6.27)	54.84	3.36 (2.22-5.11)	2.98 (1.93-4.59)	46.65	2.64 (1.99-3.49)	2.27 (1.68-3.07)

Familial Socioeconomic Status: considered as the highest level from the father and/or mother cohort member at age 10 in BCS and 11 in NCDS based on the occupational social class 1970 classification and dichotomized as Nonmanual (classes 1, II and III) and Manual (Classes IV, V and VI); Depressive symptoms during early adulthood (age 23 NCDS and 26 BCS); %: percentage with depressive symptoms; RRR: Relative Risk Ratio; 95% CI: 95% Confidence Interval. aRRR: Relative Risk Ratio adjusted for ethnicity, employment status, marital status, familial socioeconomic status, alcohol consumption, smoking status and cohort.

Frequencies of specific diseases among individuals with physical multimorbidity (pMM)

	Age 34 BCS/33 NCDS (pMM n=2,263)		Age 42 BCS/NCDS (pMM n=3,551)		Age 46 BCS/50 NCDS (pMM n=4,496)	
**Specific diseases**	**n**	**% (95% CI)**	**n**	**% (95% CI)**	**n**	**% (95% CI)**
Asthma or chronic bronchitis	611	27.00 (25.18-28.88)	1,089	30.67 (29.15-32.21)	1,382	30.74 (29.39-32.11)
Backache	1,514	66.90 (64.92-68.84)	2,254	63.48 (61.87-65.06)	2,758	61.34 (59.90-62.77)
Bladder or kidney conditions	216	9.54 (8.37-10.83)	425	11.97 (10.92-13.08)	553	12.30 (11.35-13.30)
Cancer	33	1.46 (1.01-2.04)	76	2.14 (1.69-2.67)	150	3.34 (2.83-3.90)
Cardiovascular conditions	341	15.07 (13.62-16.61)	734	20.67 (19.35-22.04)	1,391	30.94 (29.59-32.31)
Convulsions or epilepsy	170	7.51 (6.46-8.68)	265	7.46 (6.62-8.38)	313	6.96 (6.23-7.75)
Diabetes	46	2.03 (1.49-2.70)	139	3.91 (3.30-4.61)	335	7.45 (6.70-8.26)
Hearing conditions	262	11.58 (10.29-12.97)	415	11.69 (10.65-12.79)	990	22.02 (20.82-23.26)
Migraine	1,200	53.03 (50.95-55.10)	1,828	51.48 (49.81-53.13)	2,077	46.20 (44.73-47.67)
Stomach/bowel/gall conditions	698	30.84 (28.95-32.79)	1,253	35.29 (33.71-36.88)	1,598	35.54 (34.14-36.96)

pMM n: number of individuals with physical multimorbidity; %: Percentage; 95% CI: 95% Confidence Interval.

Relationship between have depressive symptoms during early adulthood (Malaise) and the development of physical multimorbidity (pMM) over adulthood taking out backache and migraine from the pMM measure.

	Age 34 BCS/33 NCDS (n=12,273)			Age 42 BCS/NCDS (n=12,022)			Age 46 BCS/50 NCDS (n=10,481)		
	**% pMM**	**RRR (95% CI)**	**aRRR (95% CI)**	**% pMM**	**RRR (95% CI)**	**aRRR (95% CI)**	**% pMM**	**RRR (95% CI)**	**aRRR (95% CI)**
pMM (dichotomous)									
No pMM (0 or 1 disease)	95.52	1.00	1.00	90.32	1.00	1.00	80.75	1.00	1.00
Yes	4.48	1.66 (1.38-1.99)	1.52 (1.24-1.87)	9.68	1.72 (1.51-1.96)	1.58 (1.38-1.81)	19.25	1.61 (1.45-1.79)	1.49 (1.32-1.67)
pMM (categorical)									
No pMM (0 or 1 disease)	95.52	1.00	1.00	90.32	1.00	1.00	80.75	1.00	1.00
2 diseases	4.00	1.56 (1.29-1.90)	1.50 (1.20-1.86)	8.13	1.60 (1.39-1.84)	1.50 (1.30-1.75)	14.48	1.51 (1.3-1.70)	1.38 (1.21-1.57)
3 diseases	0.44	2.67 (1.59-4.59)	1.81 (0.95-3.48)	1.30	2.55 (1.85-3.50)	2.23 (1.59-3.14)	3.64	1.80 (1.45-2.24)	1.73 (1.38-2.18)
4 or more diseases	0.04	2.08 (0.34-12.44)	1.41 (0.11-17.57)	0.26	2.03 (0.98-4.18)	1.26 (0.56-2.84)	1.14	2.50 (1.73-3.70)	2.12 (1.43-3.16)

Depressive symptoms during early adulthood (age 23 NCDS and 26 BCS); %: percentage with depressive symptoms; RRR: Relative Risk Ratio; 95% CI: 95% Confidence Interval. aRRR: Relative Risk Ratio adjusted for ethnicity, employment status, marital status, familial socioeconomic status, alcohol consumption, smoking status and cohort.

Cumulative incidence (%) of physical multimorbidity during childhood and adolescence (pMM) and impact on depressive symptoms at baseline

	Childhood (n=19,562)				Adolescence (n=18,212)			
	**n**	**% (95%CI)**	**RRR (95%CI)**	**aRRR (95%CI)**	**n**	**% (95%CI)**	**RRR (95%CI)**	**aRRR (95%CI)**
**pMM (dichotomous)**								
No pMM (0 or 1 disease)	18,074	92.39 (92.01-92.76)	1.00	1.00	16,263	89.30 (88.84-89.74)	1.00	1.00
Yes	1,488	7.61 (7.24-7.99)	1.79 (1.61-2.00)	1.78 (1.57-2.02)	1,949	10.70 (10.25-11.16)	1.76 (1.59-1.94)	1.66 (1.49-1.86)
**pMM (categorical)**								
No pMM (0 or 1 disease)	18,074	92.39 (92.01-92.76)	1.00	1.00	16,263	89.30 (88.84-89.74)	1.00	1.00
2 diseases	1,287	6.58 (6.24-6.94)	1.70 (1.51-1.91)	1.70 (1.49-1.95)	1,624	8.92 (8.51-9.34)	1.63 (1.47-1.81)	1.55 (1.38-1.76)
3 diseases	174	0.89 (0.76-1.02)	2.50 (1.85-3.36)	2.46 (1.75-3.48)	282	1.55 (1.35-1.74)	2.58 (2.04-3.27)	2.43 (1.86-3.18)
4 or more diseases	27	0.14 (0.09-0.20)	2.37 (1.11-5.05)	1.58 (0.65-3.84)	43	0.24 (0.17-0.32)	2.07 (1.13-3.78)	1.52 (0.77-2.99)

Childhood: age 10 in BCS and age 11 in NCDS; Adolescence: age 16 both in BCS and NCDS; 1,537 individuals that participated at baseline don't participate during childhood and 2887 individuals that participated at with information at baseline don't participate during the adolescence. Depressive symptoms at baseline assessed using the Malaise Inventory with a cut-off score 4+; n: number of individuals; RRR: Relative Risk Ratio; 95% CI: 95% Confidence Interval. aRRR: Relative Risk Ratio adjusted for gender, ethnicity, employment status, marital status, familial socioeconomic status, alcohol consumption, smoking status and cohort.

Prevalence of physical multimorbidity (pMM) at baseline overall and stratified by Cohort and by the presence of depressive symptoms during early adulthood.

	**Pooled cohorts (n= 21,099)**		**NCDS 1958 (n= 12,383)**		**BCS 1970 (n= 8,716)**	
	**n**	**% (95% CI)**	**n**	**% (95% CI)**	**n**	**% (95% CI)**
All participants						
**Depressive symptoms**	5,991	28.39 (27.79-29.01)	3,246	26.21 (25.44-27.00)	2,745	31.49 (30.52-32.48)
**pMM (dichotomous)**	4,922	23.33 (22.76-23.90)	1,658	13.39 (12.79-14.00)	3,264	37.45 (36.43-38.47)
**pMM (categorical)**						
No pMM (0 or 1 disease)	16,177	76.67 (76.10-77.24)	10,725	86.61 (86.00-87.21)	5,452	62.55 (61.53-63.57)
2 diseases	3,426	16.24 (15.74-16.74)	1,387	11.2 (10.65-11.77)	2,039	23.39 (22.51-24.30)
3 diseases	1,104	5.23 (4.94-5.54)	236	1.91 (1.67-2.16)	868	9.96 (9.34-10.61)
4 or more diseases	392	1.86 (1.68-2.05)	35	0.28 (0.20-0.38)	357	4.1 (3.69-4.53)
**Depressive symptoms *negative***						
**pMM (dichotomous)**	2,932	19.41 (18.78-20.05)	979	10.71 (10.09-11.37)	1,953	32.71 (31.51-33.92)
**pMM (categorical)**						
No pMM (0 or 1 disease)	12,176	80.59 (80.00-81.22)	8,158	89.29 (88.63-89.91)	4,018	67.29 (66.09-68.48)
2 diseases	2,164	14.32 (13.77-14.89)	833	9.12 (8.53-9.73)	1,331	22.29 (21.24-23.37)
3 diseases	615	4.07 (3.76-4.40)	132	1.44 (1.21-1.71)	483	8.09 (7.41-8.81)
4 or more diseases	153	1.01 (0.86-1.19)	14	0.15 (0.08-0.25)	139	2.33 (1.96-2.74)
**Depressive symptoms *positive***						
**pMM (dichotomous)**	1,990	33.22 (32.02-34.43)	679	20.92 (19.53-22.36)	1,311	47.76 (45.88-49.75)
**pMM (categorical)**						
No pMM (0 or 1 disease)	4,001	66.78 (65.57-67.98)	2,567	79.08 (77.64-80.47)	1,434	52.24 (50.35-54.12)
2 diseases	1,262	21.06 (20.04-22.12)	554	17.07 (15.79-18.41)	708	25.79 (24.16-27.47)
3 diseases	489	8.16 (7.48-8.88)	104	3.2 (2.63-3.87)	385	14.03 (12.75-15.38)
4 or more diseases	239	3.99 (3.51-4.51)	21	0.65 (0.40-0.99)	218	7.94 (6.96-9.02)

Depressive symptoms (age 23 NCDS and 26 BCS) assessed using the Malaise Inventory with a cut-off score 4+; n: number of individuals; % 95% CI: prevalence and 95% Confidence Interval.

Relationship between have depressive symptoms during early adulthood (Malaise) and the development of physical multimorbidity (pMM) over adulthood considering only those individuals that have reported each of the conditions to account for pMM at least two times.

	Age 34 BCS/33 NCDS30s (n=12,273)			Age 42 BCS/NCDS (n=12,022)			Age 46 BCS/50 NCDS (n=10,481)		
	**% pMM**	**RRR (95% CI)**	**aRRR (95% CI)**	**% pMM**	**RRR (95% CI)**	**aRRR (95% CI)**	**% pMM**	**RRR (95% CI)**	**aRRR (95% CI)**
pMM (dichotomous)									
No pMM (0 or 1 disease)	86.13	1.00	1.00	76.49	1.00	1.00	66.59	1.00	1.00
Yes	13.87	2.30 (2.03-2.60)	2.35 (2.02-2.73)	23.51	2.23 (2.00-2.49)	2.09 (1.86-2.36)	33.41	2.21 (1.96-2.48)	2.16 (1.90-2.46)
pMM (categorical)									
No pMM (0 or 1 disease)	86.13	1.00	1.00	76.49	1.00	1.00	66.59	1.00	1.00
2 diseases	11.28	2.09 (1.82-2.39)	2.22 (1.85-2.62)	16.86	2.00 (1.77-2.27)	1.90 (1.66-2.19)	21.94	1.74 (1.52-2.00)	1.76 (1.51-2.05)
3 diseases	2.16	3.24 (2.49-4.21)	2.92 (2.10-4.05)	5.21	2.50 (2.06-3.01)	2.33 (1.89-2.87)	8.00	2.75 (2.29-3.29)	2.61 (2.12-3.20)
4 or more diseases	0.43	4.08 (2.34-7.14)	3.65 (1.66-8.43)	1.44	4.59 (3.35-6.27)	3.72 (2.62-5.27)	3.46	4.41 (3.48-5.58)	4.19 (3.21-5.46)

Depressive symptoms during early adulthood (age 23 NCDS and 26 BCS); %: percentage with depressive symptoms; RRR: Relative Risk Ratio; 95% CI: 95% Confidence Interval. aRRR: Relative Risk Ratio adjusted for ethnicity, employment status, marital status, familial socioeconomic status, alcohol consumption, smoking status and cohort.

Loss to follow-up according to the presence of depressive symptoms during early adulthood (Malaise Inventory).

	Age 34 BCS/33 NCDS (n=12,273)		Age 42 BCS/NCDS (n=12,022)		Age 46 BCS/50 NCDS (n=10,481)	
	**n**	**%**	**n**	**%**	**n**	**%**
Without depressive symptoms at baseline (n=11,844, 74.75%)	2,609	22.03	1,204	13.04	1,525	16.87
With depressive symptoms at baseline (n=4,001, 25.25%)	963	24.07	422	13.89	599	20.08

Total number of individuals at baseline n=15,845; Depressive symptoms during early adulthood (age 23 NCDS and 26 BCS) assessed using the Malaise Inventory with a cut-off score 4+. n: number of individuals loss to follow up; %: percentage of individuals loss to follow up.

Complete models

Relationship between depressive symptoms at baseline and physical multimorbidity (dichotomous) at age 34 BCS/33 NCDS. Crude model

pMM	RRR	Std. Err.	t	P>|t|	[95% Conf.	Interval]
BD	1.751601	.0882902	11.12	0.000	1.586829	1.933483
_cons	.1931525	.0054567	-58.20	0.000	.1827483	.204149

pMM: physical multimorbidity (dichotomous) at age 34 BCS/33 NCDS; BD: Depressive symptoms at baseline; RRR: Relative Risk Ratio; Std. Err; Standard Error; t: t statistic; p>|t|: p value; 95% Conf. Interval: 95% Confidence Interval.

pMM	RRR	Std. Err.	t	P>|t|	[95% Conf.	Interval]
BD	1.672415	.0950393	9.05	0.000	1.496141	1.869459
Cohort						
BCS	.4406419	.0279127	-12.94	0.000	.3891937	.498891
Sex						
Female	1.177041	.0678046	2.83	0.005	1.051374	1.317728
Marstat						
Married	1.032345	.0601364	0.55	0.585	.9209596	1.157203
W/S/D	1.141761	.1697592	0.89	0.373	.8531328	1.528036
Alcohol						
1/2 timesweek	1.048519	.0740194	0.67	0.502	.9130324	1.204111
Less often	1.110514	.1021213	1.14	0.254	.927359	1.329842
Special ocassions	1.258478	.1242064	2.33	0.020	1.037135	1.527059
Never	1.666388	.2318505	3.67	0.000	1.26866	2.188805
Smoke						
No never	.6848695	.0426023	-6.09	0.000	.6062599	.773672
Ex smoker	.7437441	.0491208	-4.48	0.000	.6534399	.8465282
Ethnicity						
Mixed	1.621324	.7050208	1.11	0.266	.6913702	3.802146
Asian	.7713677	.2490526	-0.80	0.421	.4096619	1.452437
Black	2.382518	.8848547	2.34	0.019	1.150452	4.934055
Other	1.029761	.37291	0.08	0.935	.5063458	2.094234
Work						
Unemployed	1.154163	.1224992	1.35	0.177	.937396	1.421057
FT education	1.354763	.2238834	1.84	0.066	.9799337	1.872968
LS Sick/disabled	2.107361	.7772768	2.02	0.043	1.022772	4.342094
Housework	1.109051	.1010398	1.14	0.256	.9276896	1.325867
other situations	.7349604	.1663601	-1.36	0.174	.4716196	1.145344
SCP						
2	.9567121	.1152858	-0.37	0.714	.7552291	1.211948
3	1.021618	.1242731	0.18	0.860	.8048029	1.296844
4	1.115275	.1301322	0.94	0.350	.8872189	1.401952
5	1.068231	.1451145	0.49	0.627	.818184	1.394697
6	.9209749	.2112616	-0.36	0.720	.5854724	1.448736
_cons	.2328295	.0289416	-11.72	0.000	.1824728	.2970831

pMM: physical multimorbidity (dichotomous) at age 34 BCS/33 NCDS; BD: Depressive symptoms at baseline; Marstat: Marital status; W/S/D: Widowed, Separated or divorced; Smoke: Smoking status; Work: Employment status; FT education: Full time education; SCP: social class of the parents of the cohort member; RRR: Relative Risk Ratio; Std. Err; Standard Error; t: t statistic; p>|t|: p value; 95% Conf. Interval: 95% Confidence Interval.

Relationship between depressive symptoms at baseline and physical multimorbidity (dichotomous) at age 42 in both the BCS and the NCDS. Crude model

pMM	RRR	Std. Err.	t	P>|t|	[95% Conf.	Interval]
BD	1.758747	.0782623	12.69	0.000	1.611855	1.919026
_cons	.3602709	.0085878	-42.83	0.000	.3438263	.377502

pMM: physical multimorbidity (dichotomous) at age 42 in both BCS and NCDS; BD: Depressive symptoms at baseline; RRR: Relative Risk Ratio; Std. Err; Standard Error; t: t statistic; p>|t|: p value; 95% Conf. Interval: 95% Confidence Interval.

pMM	RRR	Std. Err.	t	P>|t|	[95% Conf.	Interval]
BD	1.628258	.0772808	10.27	0.000	1.483622	1.786995
Cohort						
BCS	.7697914	.0374928	-5.37	0.000	.6997053	.8468976
sex						
Female	1.270771	.0594374	5.12	0.000	1.159456	1.392773
marstat						
Married	1.02271	.0487393	0.47	0.637	.9315081	1.122841
W/S/D	1.215578	.1501321	1.58	0.114	.9542311	1.548503
Alcohol						
1/2 timesweek	1.004713	.056834	0.08	0.934	.899273	1.122515
Less often	1.075574	.0792159	0.99	0.323	.9309984	1.2426
Special ocassions	1.244204	.101326	2.68	0.007	1.060647	1.459528
Never	1.290297	.1542143	2.13	0.033	1.020835	1.630888
Smoke						
No never	.7770042	.039293	-4.99	0.000	.7036848	.857963
Ex smoker	.8344178	.0458016	-3.30	0.001	.7493085	.9291942
Ethnicity						
Mixed	1.163086	.4518733	0.39	0.697	.5430903	2.490872
Asian	.9933184	.23684	-0.03	0.978	.6224894	1.585057
Black	1.971274	.674505	1.98	0.047	1.007867	3.855589
Other	1.633344	.4317489	1.86	0.063	.9728953	2.742138
Work						
Unemployed	1.194659	.1059194	2.01	0.045	1.004097	1.421387
FT education	1.362995	.1851963	2.28	0.023	1.044328	1.778901
LS Sick/disabled	2.26679	.7300864	2.54	0.011	1.205736	4.261577
Housework	1.25648	.0968191	2.96	0.003	1.080353	1.461321
other situations	1.126146	.1816457	0.74	0.461	.8209083	1.544879
SCP						
2	.9905981	.1006272	-0.09	0.926	.8109915	1.209981
3	1.012366	.1042518	0.12	0.905	.8268206	1.239549
4	1.113988	.111645	1.08	0.282	.9146705	1.356739
5	1.067629	.1233385	0.57	0.572	.8504248	1.340308
6	1.059931	.1844907	0.33	0.738	.7524727	1.493017
_cons	.3509197	.0366766	-10.02	0.000	.2857942	.4308857

pMM: physical multimorbidity (dichotomous) at age 42 in both BCS and NCDS; BD: Depressive symptoms at baseline; Marstat: Marital status; W/S/D: Widowed, Separated or divorced; Smoke: Smoking status; Work: Employment status; FT education: Full time education; SCP: social class of the parents of the cohort member; RRR: Relative Risk Ratio; Std. Err; Standard Error; t: t statistic; p>|t|: p value; 95% Conf. Interval: 95% Confidence Interval.

Relationship between depressive symptoms at baseline and physical multimorbidity (dichotomous) at age 46 BCS/50 NCDS. Crude model

pMM	RRR	Std. Err.	t	P>|t|	[95% Conf.	Interval]
BD	1.668476	.0764543	11.17	0.000	1.525161	1.825259
_cons	.6617093	.0151764	-18.00	0.000	.6326226	.6921332

pMM: physical multimorbidity (dichotomous) at age 46 in the BCS and 50 in the NCDS; BD: Depressive symptoms at baseline; RRR: Relative Risk Ratio; Std. Err; Standard Error; t: t statistic; p>|t|: p value; 95% Conf. Interval: 95% Confidence Interval.

Relationship between depressive symptoms at baseline and physical multimorbidity (dichotomous) at age 46 BCS/50 NCDS. Adjusted model.

pMM	RRR	Std. Err.	t	P>|t|	[95% Conf.	Interval]
BD	1.576047	.0767987	9.34	0.000	1.432489	1.733992
Cohort						
BCS	.7471235	.0348449	-6.25	0.000	.6818571	.8186371
sex_pooled						
Female	1.098837	.0500343	2.07	0.038	1.00502	1.201412
Marstat						
Married	1.03852	.0485745	0.81	0.419	.9475488	1.138225
W/S/D	1.222848	.1575402	1.56	0.118	.949973	1.574105
Alcohol						
1/2 timesweek	1.027834	.0560983	0.50	0.615	.92356	1.143881
Less often	1.28242	.0915338	3.49	0.000	1.115	1.474979
Special ocassions	1.271909	.1042873	2.93	0.003	1.083088	1.493649
Never	1.102285	.1357196	0.79	0.429	.8659431	1.403133
Smoke						
No never	.8110678	.0404003	-4.20	0.000	.7356272	.894245
Ex smoker	.9097627	.0494054	-1.74	0.082	.817905	1.011937
Ethnicity						
Mixed	.9867707	.3800914	-0.03	0.972	.4637946	2.099456
Asian	1.240615	.3020318	0.89	0.376	.7698498	1.999253
Black	2.083229	.7496229	2.04	0.041	1.028947	4.21775
Other	1.820424	.519748	2.10	0.036	1.04021	3.185842
Work						
Unemployed	1.123918	.1027165	1.28	0.201	.9395977	1.344396
FT education	1.123967	.1514024	0.87	0.386	.8631631	1.463573
LS Sick/disabled	1.929315	.7193249	1.76	0.078	.9290426	4.006552
Housework	1.19331	.0963298	2.19	0.029	1.018684	1.39787
other situations	1.024146	.16832	0.15	0.885	.7421026	1.413382
SCP						
2	1.132509	.1073739	1.31	0.190	.9400465	1.364376
3	1.139254	.1134382	1.31	0.191	.9367309	1.385563
4	1.198152	.1156606	1.87	0.062	.991085	1.448481
5	1.214303	.1310816	1.80	0.072	.9824847	1.500818
6	1.471337	.2712171	2.09	0.038	1.0227	2.116782
_cons	.599629	.0599029	-5.12	0.000	.4928826	.7294941

pMM: physical multimorbidity (dichotomous) at age 46 in the BCS and 50 in the NCDS; BD: Depressive symptoms at baseline; Marstat: Marital status; W/S/D: Widowed, Separated or divorced; Smoke: Smoking status; Work: Employment status; FT education: Full time education; SCP: social class of the parents of the cohort member; RRR: Relative Risk Ratio; Std. Err; Standard Error; t: t statistic; p>|t|: p value; 95% Conf. Interval: 95% Confidence Interval.

Relationship between depressive symptoms at baseline and physical multimorbidity (categorical) at age 34 BCS/33 NCDS. Crude model.

pMM		RRR	Std. Err.	t	P>|t|	[95% Conf.	Interval]
No pMM		(base outcome)					
2 pMM							
	BD	1.637438	.0912968	8.84	0.000	1.46793	1.82652
	_cons	.1568475	.0048418	-60.01	0.000	.1476392	.1666302
3 pMM							
	BD	2.204542	.2368907	7.36	0.000	1.785878	2.721353
	_cons	.0299742	.0019972	-52.64	0.000	.0263046	.0341556
4 pMM							
	BD	2.435494	.5421331	4.00	0.000	1.57439	3.767574
	_cons	.0063307	.0009073	-35.32	0.000	.0047805	.0083838

pMM: physical multimorbidity (categorical) at age 34 BCS/33 NCDS; No PMM: No physical multimorbidity; 2 pMM: 2 conditons; 3 pMM: 3 conditions; 4 pMM: 4 or more conditions; BD: Depressive symptoms at baseline; RRR: Relative Risk Ratio; Std. Err; Standard Error; t: t statistic; p>|t|: p value; 95% Conf. Interval: 95% Confidence Interval.

Relationship between depressive symptoms at baseline and physical multimorbidity (categorical) at age 34 BCS/33 NCDS. adjusted model.

pMM		RRR	Std. Err.	t	P>|t|	[95% Conf.	Interval]
No pMM		(base outcome)					
2 pMM							
	BD	1.614063	.1001344	7.72	0.000	1.429266	1.822754
	Cohort						
	BCS	.5079915	.0342241	-10.05	0.000	.4451534	.5796998
	Sex						
	Female	1.177176	.0735635	2.61	0.009	1.041474	1.330559
	Marstat						
	Married	1.022624	.0647657	0.35	0.724	.9032474	1.157778
	W/S/D	1.099187	.1807247	0.58	0.565	.7963787	1.517133
	Alcohol						
	1/2 timesweek	1.086191	.0837223	1.07	0.283	.9338913	1.263328
	Less often	1.138253	.1138237	1.29	0.195	.9356625	1.38471
	Special ocassions	1.308542	.1406575	2.50	0.012	1.059962	1.615418
	Never	1.53276	.2386452	2.74	0.006	1.129653	2.079712
	Smoke						
	No never	.7566322	.051086	-4.13	0.000	.6628476	.8636861
	Ex smoker	.7995322	.0579125	-3.09	0.002	.6937145	.9214911
	Ethnicity						
	Mixed	1.530557	.726286	0.90	0.370	.603854	3.879421
	Asian	.6358695	.2429309	-1.19	0.236	.3007112	1.344579
	Black	1.704633	.7539887	1.21	0.228	.716324	4.056509
	Other	1.154972	.4315281	0.39	0.700	.5553019	2.402227
	Work						
	Unemployed	1.123681	.1313583	1.00	0.319	.8935879	1.413022
	FT education	1.315319	.2366052	1.52	0.128	.9245134	1.871325
	LS Sick/disabled	1.996967	.8012183	1.72	0.085	.9095943	4.384238
	Housework	1.034279	.1043944	0.33	0.738	.8486373	1.260531
	other situations	.6813495	.1735785	-1.51	0.132	.4135417	1.122588
	SCP						
	2	.9130971	.1175808	-0.71	0.480	.7092072	1.175603
	3	.9917256	.1316587	-0.06	0.950	.7642482	1.286911
	4	1.063509	.1348078	0.49	0.627	.8293894	1.363716
	5	1.036895	.1527679	0.25	0.806	.7763584	1.384865
	6	.9320061	.2363054	-0.28	0.782	.5641226	1.539799
	_cons	.1796993	.024234	-12.73	0.000	.137942	.2340972

3 pMM						
BD	1.88865	.2302104	5.22	0.000	1.487295	2.398312
Cohort						
BCS	.23445	.0412182	-8.25	0.000	.1661124	.3309011
Sex						
Female	1.154894	.150305	1.11	0.269	.8948729	1.490468
Marstat						
Married	1.098279	.1430868	0.72	0.472	.8507769	1.417782
W/S/D	1.234945	.378935	0.69	0.492	.6768023	2.253375
Alcohol						
1/2 timesweek	.8722953	.1357581	-0.88	0.380	.6429658	1.183421
Less often	.8886547	.1863786	-0.56	0.574	.5891261	1.340472
Special ocassions	1.068303	.2279505	0.31	0.757	.7031815	1.623011
Never	1.728236	.4859469	1.95	0.052	.9960042	2.998781
Smoke						
No never	.4569488	.066702	-5.37	0.000	.3432539	.6083027
Ex smoker	.5929968	.0843194	-3.68	0.000	.4487642	.7835856
Ethnicity						
Mixed	2.461443	1.909744	1.16	0.246	.5378096	11.26552
Asian	1.393488	.8515975	0.54	0.587	.4206408	4.616311
Black	6.654136	3.478451	3.63	0.000	2.388406	18.53852
Other	.6429487	.6578191	-0.43	0.666	.0864933	4.779365
Work						
Unemployed	1.301151	.2790423	1.23	0.220	.8546352	1.980956
FT education	1.263374	.503265	0.59	0.557	.5787033	2.758088
LS Sick/disabled	2.187993	1.673014	1.02	0.306	.4888619	9.792777
Housework	1.228128	.2331448	1.08	0.279	.8465524	1.781697
other situations	1.129754	.4848822	0.28	0.776	.4871404	2.620073
SCP						
2	1.327953	.4220393	0.89	0.372	.7117205	2.477739
3	1.440026	.4701237	1.12	0.264	.7585322	2.733798
4	1.594803	.5118028	1.45	0.146	.8489543	2.995917
5	1.26609	.4522815	0.66	0.509	.6273006	2.55537
6	1.220334	.5953527	0.41	0.683	.4684239	3.179202
_cons	.0392569	.0127168	-9.99	0.000	.0207912	.0741228
4 pMM						
BD	2.132773	.5853146	2.76	0.006	1.245495	3.652139
Cohort						
BCS	.0550396	.0400813	-3.98	0.000	.0132071	.2293733
Sex						
Female	1.346788	.4352104	0.92	0.357	.7148849	2.537246
Marstat						
Married	.9066204	.2830322	-0.31	0.754	.4916927	1.671695
W/S/D	1.451147	.8536984	0.63	0.527	.4580956	4.596919
Alcohol						
1/2 timesweek	1.184879	.5085577	0.40	0.693	.5108961	2.74799
Less often	1.988687	.9891566	1.38	0.167	.7502144	5.271661
Special ocassions	1.297028	.7046893	0.48	0.632	.4471795	3.76198
Never	5.547041	3.035383	3.13	0.002	1.897916	16.21234
Smoke						
No never	.365498	.1245582	-2.95	0.003	.1874146	.7127979
Ex smoker	.4298426	.1476389	-2.46	0.014	.219253	.8427007
Ethnicity						
Mixed	6.66e-07	.0018053	-0.01	0.996	0	.
Asian	2.011452	2.146725	0.65	0.513	.2483495	16.29131
Black	1.68e-07	.0011476	-0.00	0.998	0	.
Other	1.18e-06	.0016009	-0.01	0.992	0	.
Work						
Unemployed	1.201714	.6528466	0.34	0.735	.414203	3.486493
FT education	3.378085	2.158888	1.90	0.057	.9653075	11.82158

	RRR	Std. Err.	t	P>|t|	[95% Conf.	Interval]
LS Sick/disabled	6.569883	7.367824	1.68	0.093	.7294131	59.17547
Housework	2.377214	.8811991	2.34	0.019	1.149581	4.915829
other situations	7.14e-07	.0008871	-0.01	0.991	0	.
scp						
2	.848429	.5571667	-0.25	0.803	.2327435	3.092812
3	.4444667	.3304344	-1.09	0.277	.1027492	1.92265
4	.8344082	.5582988	-0.27	0.787	.2228177	3.124694
5	1.205354	.845656	0.27	0.790	.3019192	4.812142
6	4.42e-07	.0004194	-0.02	0.988	0	.
_cons	.0087798	.0061359	-6.78	0.000	.0022244	.0346552

pMM: physical multimorbidity (categorical) at age 34 BCS/33 NCDS; No PMM: No physical multimorbidity; 2 pMM: 2 conditons; 3 pMM: 3 conditions; 4 pMM: 4 or more conditions; BD: Depressive symptoms at baseline. Marstat: Marital status; W/S/D: Widowed, Separated or divorced; Smoke: Smoking status; Work: Employment status; FT education: Full time education; SCP: social class of the parents of the cohort member; RRR: Relative Risk Ratio; Std. Err; Standard Error; t: t statistic; p>|t|: p value; 95% Conf. Interval: 95% Confidence Interval.

Relationship between depressive symptoms at baseline and physical multimorbidity (categorical) at age 42 in both the BCS and the NCDS. Crude model.

pMM	RRR	Std. Err.	t	P>|t|	[95% Conf.	Interval]
No pMM	(base outcome)					
2 pMM						
BD	1.603636	.0814761	9.30	0.000	1.45164	1.771548
_cons	.2612491	.0070417	-49.80	0.000	.2478057	.2754217
3 pMM						
BD	1.94877	.153468	8.47	0.000	1.670043	2.274018
_cons	.0784048	.0035671	-55.96	0.000	.0717161	.0857174
4 pMM						
BD	3.001595	.3895946	8.47	0.000	2.327393	3.871101
_cons	.020617	.0017795	-44.97	0.000	.0174083	.0244171

pMM: physical multimorbidity (categorical) at age 42 in both the BCS and the NCDS; No PMM: No physical multimorbidity; 2 pMM: 2 conditons; 3 pMM: 3 conditions; 4 pMM: 4 or more conditions; BD: Depressive symptoms at baseline; RRR: Relative Risk Ratio; Std. Err; Standard Error; t: t statistic; p>|t|: p value; 95% Conf. Interval: 95% Confidence Interval.

Relationship between depressive symptoms at baseline and physical multimorbidity (categorical) at age 42 in both the BCS and the NCDS. Adjusted model.

pMM	RRR	Std. Err.	t	P>|t|	[95% Conf.	Interval]
No pMM	(base outcome)					
2 pMM						
BD	1.518524	.0819303	7.74	0.000	1.366142	1.687902
Cohort						
BCS	.8570679	.0466671	-2.83	0.005	.7703135	.9535927
Sex						
Female	1.258363	.0663664	4.36	0.000	1.134784	1.395399
Marstat						
Married	.9932107	.0535622	-0.13	0.899	.8935878	1.10394
W/S/D	1.078242	.1556712	0.52	0.602	.8125015	1.430898
Alcohol						
1/2 timesweek	.9840689	.0624629	-0.25	0.800	.8689528	1.114435
Less often	1.015694	.084473	0.19	0.851	.8629196	1.195516
Special ocassions	1.18624	.1091112	1.86	0.063	.9905541	1.420583
Never	1.244301	.1677924	1.62	0.105	.9553048	1.620724
Smoke						
No never	.8544025	.0490203	-2.74	0.006	.7635295	.956091
Ex smoker	.9279742	.0577316	-1.20	0.230	.8214486	1.048314

Ethnicity						
Mixed	1.071806	.476939	0.16	0.876	.4480654	2.563839
Asian	.9733038	.2617347	-0.10	0.920	.5745756	1.64873
Black	2.085932	.7620081	2.01	0.044	1.019287	4.26878
Other	1.820569	.5135465	2.12	0.034	1.04735	3.164627
Work						
Unemployed	1.191674	.1194165	1.75	0.080	.9791725	1.450292
FT education	1.361158	.2054239	2.04	0.041	1.012618	1.829664
LS Sick/disabled	1.671737	.6437883	1.33	0.182	.7858893	3.556103
Housework	1.154756	.1027986	1.62	0.106	.9698725	1.374884
other situations	1.035157	.1938243	0.18	0.854	.7171662	1.494144
SCP						
2	.9893503	.117014	-0.09	0.928	.7831952	1.24977
3	1.02988	.1203855	0.25	0.801	.8182889	1.296183
4	1.098481	.1237184	0.83	0.405	.8802226	1.370859
5	1.075857	.1407826	0.56	0.577	.8314155	1.392165
6	1.172253	.2264551	0.82	0.411	.8014307	1.714654
_cons	.2428961	.0288944	-11.90	0.000	.1922572	.3068728
3 pMM						
BD	1.763433	.1470086	6.80	0.000	1.49761	2.076441
Cohort						
BCS	.6121987	.057473	-5.23	0.000	.5093096	.7358731
Sex						
Female	1.253287	.1086664	2.60	0.009	1.057418	1.485436
Marstat						
Married	1.08199	.0943772	0.90	0.366	.9119623	1.283719
W/S/D	1.555534	.3075094	2.23	0.025	1.055864	2.291666
Alcohol						
1/2 timesweek	1.069351	.1141846	0.63	0.530	.86742	1.318291
Less often	1.162544	.1590034	1.10	0.271	.8891782	1.519953
Special ocassions	1.391549	.2018421	2.28	0.023	1.047208	1.849116
Never	1.189778	.2649481	0.78	0.435	.7689785	1.840846
Smoke						
No never	.6491086	.0597582	-4.69	0.000	.5419435	.7774647
Ex smoker	.6726473	.0670774	-3.98	0.000	.5532281	.8178441
Ethnicity						
Mixed	1.451921	.931052	0.58	0.561	.4128144	5.10659
Asian	.8914498	.4247073	-0.24	0.809	.3504005	2.267927
Black	2.151194	1.210663	1.36	0.173	.7138307	6.482819
Other	1.499108	.7233859	0.84	0.401	.5822101	3.859989
Work						
Unemployed	1.049298	.1726483	0.29	0.770	.7600537	1.448616
FT education	1.287937	.3292858	0.99	0.322	.7803124	2.125792
LS Sick/disabled	2.753822	1.324148	2.11	0.035	1.073095	7.066977
Housework	1.37902	.1772146	2.50	0.012	1.071975	1.774012
other situations	1.090095	.3243846	0.29	0.772	.6083707	1.953261
SCP						
2	.9003783	.1604974	-0.59	0.556	.6344977	1.277674
3	.8623214	.1587267	-0.80	0.421	.6008739	1.237528
4	1.058155	.18205	0.33	0.743	.7551311	1.482778
5	.9657337	.1909999	-0.18	0.860	.655126	1.423606
6	.6254482	.2159915	-1.36	0.175	.3173129	1.232807
_cons	.0940836	.0172747	-12.87	0.000	.0656399	.1348529
4 pMM						
BD	2.441211	.3387043	6.43	0.000	1.85997	3.20409
Cohort						
BCS	.4773518	.0848166	-4.16	0.000	.3369735	.6762097
Sex						
Female	1.502797	.2378368	2.57	0.010	1.102013	2.049341
Marstat						

	RRR	Std. Err.	t	P>|t|	[95% Conf.	Interval]
Married	1.152742	.1779041	0.92	0.357	.8518537	1.559909
W/S/D	1.482211	.4852389	1.20	0.229	.7802755	2.815608
Alcohol						
1/2 timesweek	1.042002	.215645	0.20	0.842	.6945347	1.563304
Less often	1.534695	.3718363	1.77	0.077	.9544875	2.467595
Special ocassions	1.426744	.3746197	1.35	0.176	.8527235	2.387174
Never	2.196096	.7086728	2.44	0.015	1.166715	4.13369
Smoke						
No never	.5331353	.088158	-3.80	0.000	.3855536	.7372082
Ex smoker	.5933428	.1033681	-3.00	0.003	.4217113	.8348265
Ethnicity						
Mixed	1.113524	1.176403	0.10	0.919	.1404208	8.830142
Asian	1.559352	.9706786	0.71	0.475	.4602686	5.282953
Black	9.77e-07	.0014008	-0.01	0.992	0	.
Other	9.82e-07	.0010388	-0.01	0.990	0	.
Work						
Unemployed	1.771247	.4258631	2.38	0.017	1.105662	2.837501
FT education	1.671802	.7841838	1.10	0.273	.6666765	4.19232
LS Sick/disabled	6.805885	3.652501	3.57	0.000	2.377232	19.48487
Housework	1.694347	.3399721	2.63	0.009	1.143426	2.510711
other situations	2.347417	.8953679	2.24	0.025	1.111525	4.957485
SCP						
2	1.630075	.7143988	1.11	0.266	.6869071	3.868273
3	1.678996	.7553694	1.15	0.251	.6908854	4.08031
4	1.783523	.8005735	1.29	0.200	.7340705	4.333309
5	1.622816	.7772087	1.01	0.314	.629784	4.181641
6	1.958742	1.138361	1.16	0.249	.6228366	6.159997
_cons	.0120994	.0054766	-9.75	0.000	.0049622	.0295022

pMM: physical multimorbidity (categorical) at age 42 in both the BCS and the NCDS; No PMM: No physical multimorbidity; 2 pMM: 2 conditons; 3 pMM: 3 conditions; 4 pMM: 4 or more conditions; BD: Depressive symptoms at baseline. Marstat: Marital status; W/S/D: Widowed, Separated or divorced; Smoke: Smoking status; Work: Employment status; FT education: Full time education; SCP: social class of the parents of the cohort member; RRR: Relative Risk Ratio; Std. Err; Standard Error; t: t statistic; p>|t|: p value; 95% Conf. Interval: 95% Confidence Interval.

Relationship between depressive symptoms at baseline and physical multimorbidity (categorical) at age 46 BCS/50 NCDS. Crude model.

pMM	RRR	Std. Err.	t	P>|t|	[95% Conf.	Interval]
No pMM	(base outcome)					
2 pMM						
1.depre26_pooled	1.419426	.0766538	6.49	0.000	1.276865	1.577904
_cons	.4206116	.0111876	-32.56	0.000	.3992461	.4431206
3 pMM						
1.depre26_pooled	1.900615	.1320322	9.24	0.000	1.658681	2.177838
_cons	.1698785	.0064521	-46.67	0.000	.1576919	.1830069
4 pMM						
1.depre26_pooled	2.585617	.2378306	10.33	0.000	2.159081	3.096418
_cons	.0712191	.0039976	-47.07	0.000	.0637996	.0795014

pMM: physical multimorbidity (categorical) at age 46 BCS/50 NCDS; No PMM: No physical multimorbidity; 2 pMM: 2 conditons; 3 pMM: 3 conditions; 4 pMM: 4 or more conditions; BD: Depressive symptoms at baseline; RRR: Relative Risk Ratio; Std. Err; Standard Error; t: t statistic; p>|t|: p value; 95% Conf. Interval: 95% Confidence Interval.

Relationship between depressive symptoms at baseline and physical multimorbidity (categorical) at age 46 BCS/50 NCDS. Adjusted model.

pMM	RRR	Std. Err.	t	P>|t|	[95% Conf.	Interval]
No pMM	(base outcome)					
2 pMM						
BD	1.376608	.0785102	5.60	0.000	1.231019	1.539415
Cohort						
BCS	.8303553	.0445543	-3.46	0.001	.7474654	.9224372
Sex						
Female	1.108801	.0582992	1.96	0.049	1.000227	1.229161
Marstat						
Married	1.019875	.0552043	0.36	0.716	.9172182	1.134022
W/S/D	1.20684	.1789406	1.27	0.205	.9024853	1.613836
Alcohol						
1/2 timesweek	1.048385	.0663054	0.75	0.455	.92616	1.186739
Less often	1.280573	.1050955	3.01	0.003	1.090303	1.504049
Special ocassions	1.205316	.1152503	1.95	0.051	.9993332	1.453756
Never	.9755748	.1442504	-0.17	0.867	.73013	1.30353
Smoke						
No never	.9299513	.0539777	-1.25	0.211	.8299528	1.041998
Ex smoker	1.028834	.0650382	0.45	0.653	.9089419	1.16454
Ethnicity						
Mixed	.93674	.4295004	-0.14	0.887	.3813602	2.300927
Asian	1.277443	.3571474	0.88	0.381	.7385144	2.209653
Black	1.756592	.7217976	1.37	0.170	.7850537	3.930453
Other	1.726718	.5651802	1.67	0.095	.9090043	3.280024
Work						
Unemployed	1.051312	.1133392	0.46	0.643	.8510707	1.298666
FT education	1.102112	.1711869	0.63	0.531	.8128509	1.494309
LS Sick/disabled	1.357645	.6261821	0.66	0.507	.5497665	3.352695
Housework	1.059741	.1012369	0.61	0.544	.8787861	1.277958
other situations	.9104036	.1794429	-0.48	0.634	.6186668	1.339711
SCP						
2	1.240244	.1445352	1.85	0.066	.9857499	1.560441
3	1.237239	.1522969	1.73	0.085	.9703938	1.577463
4	1.253274	.1478431	1.91	0.057	.9933294	1.581244
5	1.20311	.1564242	1.42	0.155	.9319847	1.553108
6	1.658603	.3712386	2.26	0.026	1.063532	2.586629
_cons	.3255915	.0398748	-9.16	0.000	.2559095	.4142473
3 pMM						
BD	1.774314	.1311091	7.76	0.000	1.535086	2.050823
Cohort						
BCS	.672139	.0513665	-5.20	0.000	.5786396	.7807464
Sex						
Female	1.087387	.0795815	1.14	0.252	.9420802	1.255105
Marstat						
Married	1.106834	.0824478	1.36	0.173	.9564817	1.280821
W/S/D	1.195654	.2395821	0.89	0.373	.8073156	1.770794
Alcohol						
1/2 timesweek	.9364221	.0820958	-0.75	0.454	.788582	1.111979
Less often	1.220534	.1373565	1.77	0.077	.9789416	1.521749
Special ocassions	1.230066	.1555764	1.64	0.102	.9599971	1.576111
Never	.789191	.1680445	-1.11	0.266	.5199163	1.197928
Smoke						
No never	.6922311	.0550334	-4.63	0.000	.5923513	.8089523
Ex smoker	.8121073	.0687157	-2.46	0.014	.6880021	.9585991
Ethnicity						
Mixed	.6093199	.4693885	-0.64	0.520	.1344684	2.761027

	RRR	Std. Err.	t	p>|t|	95% Conf. Interval	
Asian	1.15409	.4631408	0.36	0.721	.5255919	2.53414
Black	3.905882	1.709743	3.11	0.002	1.655692	9.214226
Other	1.800847	.7533795	1.41	0.160	.7931807	4.088665
Work						
Unemployed	1.005226	.148498	0.04	0.972	.7525219	1.342791
FT education	1.320547	.269716	1.36	0.173	.8849072	1.970652
LS Sick/disabled	1.825104	.9920806	1.11	0.268	.6289181	5.296406
Housework	1.218587	.1474893	1.63	0.102	.9612426	1.544829
other situations	.9746796	.2631449	-0.09	0.924	.574183	1.654525
SCP						
2	.9314973	.1424053	-0.46	0.643	.6894248	1.258567
3	.9204435	.14326	-0.53	0.595	.6779239	1.249722
4	1.027068	.1516459	0.18	0.857	.7686032	1.37245
5	1.18173	.1946345	1.01	0.311	.8553657	1.632617
6	1.123414	.316242	0.41	0.680	.6453495	1.955619
_cons	.2108189	.0323995	-10.13	0.000	.1559499	.2849929
4 pMM						
BD	2.271413	.2235451	8.34	0.000	1.872939	2.754664
Cohort						
BCS	.5172686	.0595718	-5.72	0.000	.4127495	.6482548
Sex						
Female	1.068924	.1134565	0.63	0.530	.8681595	1.316116
Marstat						
Married	.985964	.1049598	-0.13	0.894	.8002895	1.214717
W/S/D	1.313935	.328431	1.09	0.275	.8050212	2.14457
Alcohol						
1/2 timesweek	1.164278	.1575235	1.12	0.261	.8930776	1.517833
Less often	1.487997	.251704	2.35	0.019	1.068104	2.07296
Special ocassions	1.786616	.3170831	3.27	0.001	1.261705	2.529907
Never	2.644875	.5799893	4.44	0.000	1.720863	4.065033
Smoke						
No never	.5757454	.0646138	-4.92	0.000	.4620652	.7173939
Ex smoker	.6447673	.0772924	-3.66	0.000	.509758	.8155338
Ethnicity						
Mixed	1.781727	1.055583	0.97	0.330	.557886	5.690321
Asian	1.336819	.6243587	0.62	0.534	.5351656	3.339314
Black	2.33e-06	.0014057	-0.02	0.983	0	.
Other	2.381563	1.216883	1.70	0.089	.8748439	6.483262
Work						
Unemployed	1.746571	.29265	3.33	0.001	1.257653	2.425558
FT education	.7485604	.2965827	-0.73	0.465	.3443318	1.627334
LS Sick/disabled	4.714962	2.349828	3.11	0.002	1.775217	12.5229
Housework	1.758506	.2645866	3.75	0.000	1.309393	2.361663
other situations	1.805157	.540751	1.97	0.049	1.003529	3.247133
SCP						
2	1.111686	.2713166	0.43	0.665	.6880676	1.796112
3	1.235107	.3102334	0.84	0.401	.7536298	2.024188
4	1.380315	.3447526	1.29	0.198	.8435889	2.258528
5	1.342902	.3555202	1.11	0.266	.7981669	2.259409
6	1.480293	.5540815	1.05	0.295	.7093195	3.089253
_cons	.064713	.0163697	-10.82	0.000	.0393779	.1063481

pMM: physical multimorbidity (categorical) at age 46 BCS/50 NCDS; No PMM: No physical multimorbidity; 2 pMM: 2 conditons; 3 pMM: 3 conditions; 4 pMM: 4 or more conditions; BD: Depressive symptoms at baseline. Marstat: Marital status; W/S/D: Widowed, Separated or divorced; Smoke: Smoking status; Work: Employment status; FT education: Full time education; SCP: social class of the parents of the cohort member; RRR: Relative Risk Ratio; Std. Err; Standard Error; t: t statistic; p>|t|: p value; 95% Conf. Interval: 95% Confidence Interval.
